# Phenolic Profile and Bioactive Prospects of Wild *Annona* Species From Angola

**DOI:** 10.1002/cbdv.202503294

**Published:** 2025-12-10

**Authors:** Josefa Rangel, Ângela Liberal, Tiane C. Finimundy, Tânia S. P. Pires, Pedro Cravo, Maria Conceição Silva, Gustavo Capatti Cassiano, Lillian Barros, Maria M. Romeiras, Ângela Fernandes

**Affiliations:** ^1^ Linking Landscape, Environment, Agriculture and Food Research Center (LEAF), Associated Laboratory TERRA, Instituto Superior de Agronomia (ISA) Universidade de Lisboa Lisboa Portugal; ^2^ Centro de Botânica Universidade Agostinho Neto Luanda Angola; ^3^ CIMO, LA SusTEC Instituto Politécnico de Bragança, Campus de Santa Apolónia Bragança Portugal; ^4^ Global Global Health and Tropical Medicine (GHTM) Associate Laboratory in Translation and Innovation Towards Global Health, (LA‐REAL) Instituto de Higiene e Medicina Tropical, (IHMT), Universidade NOVA De Lisboa (UNL) Lisboa Portugal; ^5^ Centre For Ecology Evolution and Environmental Changes (cE3c) & CHANGE— Global Change and Sustainability Institute Faculdade de Ciências, Universidade De Lisboa Lisboa Portugal

**Keywords:** African useful flora, Annonaceae, antimalarial, antimicrobial, antioxidant, phenolic compounds

## Abstract

*Annona* species (Annonaceae family) are valued for their nutritional and medicinal importance, especially in traditional medicine. This study investigated the phenolic profiles of the Angolan *Annona muricata*, *Annona squamosa*, and *Annona senegalensis* leaves, stem barks, and seeds hydroethanolic, infusion, and decoction extracts, also evaluating their antioxidant, antimalarial, and antimicrobial potential. Our results showed a vibrant phenolic profile in all the studied species, with *A. muricata* standing out with 44 compounds identified, with leaves containing the highest concentration of total phenolic compounds, particularly in *A. senegalensis*. Procyanidin trimer was the primary compound found in *A. muricata* leaf and stem bark hydroethanolic extract and infusion preparation, while in *A. squamosa*, epigallocatechin and catechin prevail. In *A. senegalensis*, quercetin‐3‐*O*‐rutinoside was primarily detected in the leaves and isorhamnetin‐3‐*O*‐rutinoside in the stem barks. Extracts from *A. senegalensis* performed a higher antioxidant capacity, while the hydroethanolic extract of *A. muricata* displayed better antimalarial activity. *A. senegalensis* showed the highest antioxidant activity, while *A. muricata* extract was most effective against malaria; all extracts displayed antibacterial effects. These results reveal the remarkable phenolic richness and bioactive potential of the studied species, supporting their traditional medicinal uses and emphasizing the pharmaceutical and industrial relevance of *Annona* species.

## Graphical abstract

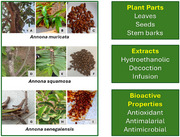

## 1 Introduction

Useful plants are fundamental to rural African communities, providing food, medicines, and other essential resources, and thus representing a critical ecosystem provisioning service [1]. The traditional knowledge associated with their uses constitutes valuable cultural heritage but is increasingly threatened by erosion and intergenerational loss [2]. Although rural populations retain substantial empirical expertise, scientific validation is required to safeguard this knowledge and to unlock the therapeutic, nutritional, and bioactive potential embedded in African flora. Angola, the largest country in southern Africa, harbors a wide range of ecosystems and habitats, spanning from the rainforests of Cabinda to the Namibe Desert [3]. This ecological heterogeneity underpins a remarkably rich flora, with numerous species traditionally employed by rural communities for food [4] and medicinal purposes [5]. For many of these communities, indigenous plants remain essential as primary or complementary therapeutic resources. Such practices have historically played a central role in addressing diverse health conditions [6] and continue to be vital for human well‐being and survival.

Within Angola's flora diversity, the Annonaceae family is particularly noteworthy due to its nutritional and ethnomedicinal importance, contributing simultaneously to food security and traditional healthcare systems [7]. However, despite its relevance, this family remains significantly understudied, underscoring the need for comprehensive ethnobotanical and pharmacological investigations in this country.

The Annonaceae family comprises ca. 2500 species and is widespread in tropical regions, encompassing 108 recognized genera [8]. The genus *Annona* are native to Central America and the West Indies and are distributed across various continents, such as Asia, South and Central America, Australia, and Africa, occurring mainly in tropical regions and arboreal biomes [9]. These include several tropical and sub‐tropical cultures, some with high global relevance at a socioeconomic level [10], providing a wide variety of edible and nonedible plant species, highly relevant at a nutritional and medicinal level [11]. Recent studies have shown that extracts from different plant structures (roots, peel, stem bark, and others) of *Annona* species are widely used in traditional medicine to treat several diseases [7, 12]. A wide range of phytochemical constituents, including acetogenins, alkaloids, phenolic compounds, essential oils, cyclopeptides, carotenoids, amino acids, anthocyanins, vitamins, and minerals has been identified [13]. These compounds are responsible for various biological activities, such as cytotoxic, antitumor, antiparasitic, immunosuppressive, antimicrobial, antidiabetic, and pesticide activities, among others [14]. However, the chemical composition of *Annona* genus plants varies significantly between different species, making individual investigation of their specific bioactive potential necessary [13].

*Annona muricata* L., traditionally known as soursop or graviola, is a small tree whose edible fruits are used to produce sweets, juices, and ice creams. Among the *Annona* genus, only *A. muricata* is used as a nutraceutical, with its aerial parts commonly used as a food supplement [15]. Some of the pharmacological properties attributed to this species include vasodilatory, antiviral, antimutagenic, and antihypertensive effects, which are directly related to the presence of bioactive compounds such as acetogenins, terpenoids, flavonoids, coumarins, anthraquinones, and others [16]. *Annona squamosa* L., in turn, commonly known as custard apple or sugar apple, is a small tropical tree or shrub whose bark, leaves, fruits, and seeds are traditionally used by different ethnic communities as a natural medicine and in various food options [4]. The fruits are rich in vitamin C, dietary fiber, vitamin B1 (thiamine), and potassium [17], while extracts obtained from other sections of the plant (bark, roots, leaf, stem, seeds) have been used to treat dysentery, epilepsy, hemorrhages, fever, and tumors [10]. Studies have reported the occurrence of several compounds, such as glycosides, phytosterols, saponins, tannins, alkaloids, flavonoids, acetogenins, among others, which are responsible for the extensive biological properties of this species [17]. Finally, *Annona senegalensis* Pers., known as wild custard or wild soursop, is one of the most reported and used species in several African countries, mainly for its therapeutic potential. Leaves of *A. senegalensis*, for example, are commonly used in the treatment of tuberculosis, yellow fever, and smallpox, while a decoction of the bark of the stem is used to treat injuries caused by poisonous animals, and the roots in reproductive problems, and to treat malaria and diabetes [18].

Despite the important contribution of recent studies in characterizing the phenolic profile and bioactive potential of different *Annona* species, there is still a large scientific gap regarding specific species and the influence of the type of extraction/solvents used in the detection of these compounds. Thus, the objective of this study is to provide a comprehensive assessment of the phenolic profile of Angolan *A. muricata*, *A. squamosa*, and *A. senegalensis* leaves, stem bark, and seeds, using hydroethanolic extracts, decoction, and infusion preparations. In addition, their antioxidant, antimalarial, and antimicrobial potential was evaluated to support their use in traditional medicine and promote the exploitation of bioactive compounds of interest for various industries.

## 2 Results and Discussion

### 2.1 Phenolic Compounds Identification and Quantification

The phenolic composition of *A. muricata*, *A. squamosa*, and *A. senegalensis* was studied using hydroethanolic extracts, decoctions, and infusions of stem bark, leaves, and seeds. For each compound, the analysis included retention time, *λ*_max_, pseudo‐molecular ions, major fragment ions in MS2, and tentative identification, as detailed in Tables SM1–SM3.

The individual phenolic compounds in these species were tentatively identified based on the data presented and, when possible, in parallel with existing standard compounds and/or published literature. The results attained for *A. muricata* hydroethanolic extracts, decoction and infusion preparations are presented in supplementary material (Table SM1). A total of 44 compounds were identified, including 8 phenolic acids, 11 condensed tannins (polymeric procyanidins), 5 flavan‐3‐ols, 2 flavanones, 1 flavone, 4 flavonols, and 3 acetogenins. Twenty‐seven compounds were tentatively identified as flavonoids, more precisely described as follows.

Procyanidins polymeric stands out, corresponding to 11 of the total detected compounds (Peaks **8**, **11**, **17**, **22**, **25**, **26**–**28**, **30**, **32**, **33**). Compounds **11** and **17** presented deprotonated pseudo‐molecular ions [M−H]^−^ at *m*/*z* 577 and daughter ions at *m*/*z* 451, 425, 407, and 289, which allowed their identification as procyanidin dimer isomers. Compounds **22**, **27**, **28**, and **33** ([M−H]^−^ at *m*/*z* 865), in turn, coincided with procyanidin trimer isomers, while compounds **8**, **25**, **32** ([M−H]^−^ at *m*/*z* 1153) and compounds **26** and **30** ([M−H]^−^ at *m*/*z* 1441), were tentatively identified as procyanidin tetramer and pentamer isomers, respectively. Procyanidin oligomers (dimers, tetramers, and pentamers) are differentiated using mass spectrometry techniques based on their distinct molecular ions and fragmentation patterns. In mass spectra, molecular ions increase by 288 *m*/*z* units for each additional monomer unit. Thus, fragmentation patterns show sequential losses of 288 u, corresponding to monomeric units. Some have also been identified by Ochoa‐Jiménez et al. [19], who studied a metabolomic approach for identifying phenolic compounds in *A. muricata* fruits. Procyanidins are notably multifunctional, a characteristic derived from the unique chemical structure of their monomeric units. Focusing specifically on procyanidins and prodelphinidins, these compounds are based on (*epi*)catechin and (*epi*)gallocatechin. Each of these molecules consists of a B ring containing catechol or pyrogallol groups, an A ring resembling resorcinol, and a heterocyclic C ring.

Quercetin *O*‐glycosidic bonds appear next, with four compounds identified (Peaks **35**–**37** and **41**), whose differences are linked to the type of sugar(s) bound. Compound **35** was tentatively identified as quercetin‐3‐*O*‐(6‐*O*‐rhamnosyl)‐galactoside, presenting a pseudo‐molecular ion [M−H]^−^ at *m*/*z* 609 and a single MS^2^ fragment at 301, corresponding to quercetin bound to a disaccharide of rhamnose and galactose. Quercetin‐3‐*O*‐rutinoside (Peak **36**), in turn, typically shows a molecular ion [M−H]^−^ at *m*/*z* 609 in mass spectrometry. The MS2 fragmentation yielding *m*/*z* 301 represents the quercetin aglycone after losing the rutinose moiety (308 u). Rutinose is cleaved from the molecule during fragmentation. Additional characteristic fragments like *m*/*z* 300 and *m*/*z* 151 from quercetin fragmentation were also observed. This fragmentation pattern is consistent with quercetin‐3‐*O*‐rutinoside's structure, where the rutinose disaccharide is cleaved to yield the quercetin aglycone at *m*/*z* 301. Compounds **37** and **41** ([M−H]^−^ at *m*/*z* 463 and 447, respectively, at *m*/*z* 301), in turn, were tentatively identified as quercetin‐3‐*O*‐glucoside and quercetin‐3‐*O*‐rhamnoside, respectively, being bound with a single sugar. Mancini et al. [20] were also able to identify different quercetin derivatives in the aqueous extract of *A. muricata* leaves, among them quercetin‐3‐*O*‐glucoside and quercetin‐3‐*O*‐rhamnoside, and others also bound to simple or complex sugars. Compounds **18** and **20** were tentatively identified with pure standards of epicatechin as (−)‐epicatechin hexoside ([M−H]^−^ at *m*/*z* 431 and *m*/*z* 289), and (−)‐epicatechin ([M−H]^−^ at *m*/*z* 289 and *m*/*z* 245). Compounds **5** and **19**, in turn, were identified as dihydroxygallocatechin and epigallocatechin, respectively, where the first present a pseudo‐molecular ion [M−H]^−^ at *m*/*z* 341 and fragments at *m*/*z* 305 and 169, which correspond to the loss of one hydroxyl group on the B‐ring compared to epigallocatechin ([M−H]^−^ at 305 and *m*/*z* 219, 179, and 125). Epicatechin derivatives were also identified in *A. muricata* by other authors [19, 20, 21].

Two isorhamnetin glycosylated were also identified in *A. muricata* extracts, namely, isorhamnetin‐3‐*O*‐deoxyhexosyl hexoside and isorhamnetin‐3‐*O*‐rutinoside (Peak **34** and **40**; [M−H]^−^ at *m*/*z* 623), both with a single fragment at *m*/*z* 315. As these share the same molecular mass, resulting in identical *m*/*z* 623 [M−H]^−^ ions in negative ion mode mass spectrometry, detailed MS/MS fragmentation analysis, focusing on intermediate fragments and their intensities were required. Additional techniques, such as comparison of retention times with authentic standards and complementary methods were performed. Isorhamnetin derivatives were identified for the first time in *A. muricata* by Abdalla et al. [22], who based their identification on previous published data from other species [23].

Kaempferol derivatives were likewise detected in the analyzed extracts with kaempferol‐3‐*O*‐(6‐*O*‐thamnosyl)‐glucoside and kaempferol‐3‐*O*‐rutinoside (Peaks **38** and **39**), which presented a pseudo‐molecular ion [M−H]^−^ at *m*/*z* 593 and a single fragment at *m*/*z* 285, these being earlier identified by other authors in this and other Annonaceae species [19, 22]. Other identified flavonoids include naringenin hexoside pentoside (Peak **16**; [M−H]^−^ at *m*/*z* 565 and *m*/*z* 271), chrysin‐6‐*C*‐pentoside‐8‐*C*‐glucoside (Peak **23**; [M−H]^−^ at 547 and *m*/*z* 253), and eriodictyol‐rutinoside (Peak **31**; [M−H]^−^ at *m*/*z* 595 and *m*/*z* at 287 and 269). Despite these already been identified in Annonaceae species [24, 25, 26], at the best of our knowledge, this is the first report of the described compounds in *A. muricata*.

Regarding phenolic acids, 14 compounds were tentatively identified in *A. muricata* hydroethanolic extracts, decoction and infusion preparations, of which caffeic and ferulic acid derivatives stand out. Compounds **2**, **3**, and **24** were positively identified by comparison with commercial standards as caffeic acid hexoside, dihydrocaffeic acid glucoronide, and methyl‐sulfate caffeic acid hexoside, presenting deprotonated pseudo‐molecular ions [M−H]^−^ at *m*/*z* 341 (*m*/*z* 179), 391 (*m*/*z* 217, 179, and 135), and 437 (*m*/*z* 341 and 179), respectively. Other similar compounds were identified in *Annona* species, and specifically in *A. muricata*, namely, 3‐*O*‐caffeic‐quinic acid derivatives [20] and caffeic acid [12]. In parallel, 4‐*O*‐caffeoylquinic acid (Peak **13**; [M−H]^−^ at *m*/*z* 353 and *m*/*z* 191, 179, and 173), an ester of caffeic and quinic acid, was identified in *A. muricata*. Compounds **6**, **9**, and **15** presented MS^2^ fragments at *m*/*z* 193 (Peak **6**) and 191 (Peak **9** and **15**) and a characteristic UV spectrum of ferulic acid derivatives, with a pseudo‐molecular ion [M−H]^−^ at *m*/*z* 355, being tentatively identified as ferulic acid hexoside, ferulic acid‐*O*‐hexoside, and ferulic acid‐6‐*C*‐hexoside based on authentic standards. Ferulic acid hexoside was previously identified in *A. muricata* by other authors [21]. In the studied hydroethanolic extracts, decoction and infusion preparations, 3,4‐di‐*p*‐coumaroylquinic acid (Peak **7**) and *p*‐coumaroylquinic acid (Peak **12**) were also identified, each one presenting different pseudo‐molecular ions ([M−H]^−^ at *m*/*z* 675 and 337, respectively) and daughter fragment ions, and whose key differences lie on the number of *p*‐coumaric acid groups attached to the quinic acid backbone. Galloyl‐*p*‐coumaric acid was also identified (Peak **4**; [M−H]^−^ at *m*/*z* 315 and a fragment ion at *m*/*z* 163). Ochoa‐Jiménez et al. [19] identified several coumaric acid derivatives in *A. muricata* extracts, namely, two coumaric acid hexosides and coumaric and *p*‐coumaric acids, reinforcing their positive identification in this species. Other single phenolic acids were identified in *A. muricata*, namely, glucaric (Peak **1**; [M−H]^−^ at *m*/*z* 209) and quinic (Peak **10**; [M−H]^−^ at *m*/*z* 191) acids, as well as shikimic acid‐*O*‐hexoside (Peak **21**; [M−H]^−^ 337), and 3‐*O*‐methyl gallic acid sulfate hexoside (Peak **29**; [M−H]^−^ at *m*/*z* 425), these being identified in comparison with commercial standards, and previously detected by other authors [19, 20, 22]. Acetogenins (Peaks **42**–**44**) are usually described as lipophilic neurotoxins [27]. Our results suggested the presence of three acetogenin isomers in *A. muricata* extracts. The mass spectrum of these presented a major ion peak at *m*/*z* 565, corresponding to the [M−H]^−^ at *m*/*z* and a pseudo‐molecular fragmentation pattern *m*/*z* 521, 381, 311, 193 in the isomer I, and *m*/*z* 521 for isomers II and III. Abdallah et al. [28] found that acetogenins were the primary class of compounds found in both ethanolic and aqueous extracts of *A. muricata*, having identified 36 new acetogenins for the first time in this species. In addition, in this study, the authors collected data on the quantity of the total acetogenins found in other investigations with *A. muricata* [21, 29, 30, 31], which sum 106 compounds to those previously detected. Despite acetogenins in *A. muricata* being associated with atypical Parkinsonism and neurodegeneration (rats) [30], in other *Annona* species, cytotoxic effects against different types of cancer cells were revealed, improved by the presence of flavonoids to enable maximum therapeutic effects [29], as well as with antidepressant and antidiabetic effects in rats [32].

Data regarding the quantification of the phenolic compounds identified in *A. muricata* stem bark, leaves, and seeds extracts (Figure S1), are presented in Table 1. Among the analyzed plant parts, leaves stand out, with all the studied extracts presenting the highest concentration in total phenolic compounds (7.25, 9.12, and 8.96 mg/g extract, respectively, for hydroethanolic extracts, decoction and infusion preparations). In these, procyanidin trimer (Peak **22**) and chrysin‐6‐*C*‐pentoside‐8‐*C*‐glucoside (Peak **23**) appear as the major compounds, with concentrations ranging from 1.03 to 1.25 mg/g extract for the first, and 0.91–1.078 mg/g extract for the latest. These compounds were also found in the hydroethanolic extracts and infusion preparations of the stem bark of *A. muricata* in considerable amounts, likely part of the major identified compounds in this plant structure. Over the years, some studies have investigated the bioactive potential of procyanidins, which showed antioxidant [12, 33], anticancer [34, 35, 36], hypoglycemic [37, 38], among other biological activities, and which physiological functions may vary depending on their structure and degree of polymerization [39]. Chrysin, in turn, had few reports in the current literature, given their limited bioavailability and absorption. However, this had been described as a potent inhibitor of the activation of human immunodeficiency virus (HIV) in models of latent infection [40], showing also some anti‐inflammatory, antidiabetic, antioxidant, and anticancer effects [41, 42, 43]. On *A. muricata* stem barks, other compounds also stand out, namely, epigallocatechin (Peak **19**) and (−)‐epicatechin (Peak **20**), in all the studied extracts, as well as shikimic acid‐*O*‐hexoside (Peak **21**) and methyl‐sulfate caffeic acid hexoside (Peak **24**) only in infusion, with concentrations varying from 0.460 to 0.466 mg/g extract. Recent studies have been suggested the bioactive potential of epicatechin, pointing this as a prominent antioxidant and anti‐inflammatory compound that also enhances muscle performance, improves symptoms of cardiovascular and cerebrovascular diseases, prevents diabetes, and protects the nervous system [44]. Epigallocatechin, in turn, has been associated with anti‐atherosclerosis mechanisms [45], and used as a nutraceutical to target metabolomic syndromes beyond antioxidant and anti‐inflammatory properties [46]. Regarding seeds, besides the compounds **18**, **19**, and **24** previously described, procyanidin tetramer (Peak **25**) was also detected in similar amounts (0.461–0.467 mg/g extract). Among all the studied *A. muricata* plant structures, seed extracts displayed the lowest quantity of total phenolic compounds, ranging from 1.146 to 2.709 mg/g extract, with only flavonoids identified. Moreover, decocted preparations, except from leaves, presented the least sum of total compounds.

**TABLE 1:** Content (mg/g extract) of the phenolic compounds identified in the leaves, stem bark, and seeds of *Annona muricata* hydroethanolic extracts, decoction, and infusion preparations (mean ± SD, *n* = 3).

	Leaves			Stem bark			Seeds		
Peaks	Hydroethanolic	Decoction	Infusion	Hydroethanolic	Decoction	Infusion	Hydroethanolic	Decoction	Infusion
**1**	0.085 ± 0.001	0.35 ± 0.01	0.288 ± 0.01	0.067 ± 0.001	0.069 ± 0.002	0.267 ± 0.01	nd	nd	nd
**2**	0.041 ± 0.004	0.196 ± 0.01	0.04 ± 0.01	traces	traces	traces	traces	nd	traces
**3**	0.028 ± 0.001	0.107 ± 0.002	0.19 ± 0.01	0.056 ± 0.001	nd	nd	nd	nd	nd
**4**	0.062 ± 0.001	0.193 ± 0.002	0.21 ± 0.01	0.031 ± 0.001	0.031 ± 0.001	0.042 ± 0.005	nd	nd	nd
**5**	0.13 ± 0.002	0.212 ± 0.005	0.236 ± 0.005	nd	nd	nd	nd	nd	nd
**6**	0.043 ± 0.001	0.26 ± 0.01	0.242 ± 0.005	nd	nd	nd	nd	nd	nd
**7**	0.051 ± 0.001	0.69 ± 0.02	0.88 ± 0.01	nd	nd	nd	nd	nd	nd
**8**	0.144 ± 0.003	0.107 ± 0.001	0.144 ± 0.003	nd	nd	nd	nd	nd	nd
**9**	0.08 ± 0.001	0.195 ± 0.005	0.176 ± 0.001	nd	nd	nd	nd	nd	nd
**10**	0.044 ± 0.001	nd	0.063 ± 0.001	nd	nd	nd	nd	nd	nd
**11**	traces	traces	traces	nd	nd	nd	nd	nd	nd
**12**	0.136 ± 0.004	0.26 ± 0.01	0.213 ± 0.004	0.06 ± 0.01	nd	nd	0.061 ± 0.001	0.047 ± 0.001	0.073 ± 0.001
**13**	0.164 ± 0.005	0.33 ± 0.01	0.172 ± 0.003	nd	0.033 ± 0.001	0.038 ± 0.001	nd	nd	nd
**14**	0.46 ± 0.01	0.41 ± 0.01	0.42 ± 0.01	0.143 ± 0.003	0.58 ± 0.02	0.5 ± 0.02	0.049 ± 0.01	0.049 ± 0.001	0.042 ± 0.001
**15**	0.07 ± 0.002	0.119 ± 0.004	0.076 ± 0.002	nd	nd	nd	nd	nd	nd
**16**	0.14 ± 0.01	0.093 ± 0.003	0.2 ± 0.01	nd	nd	nd	traces	nd	traces
**17**	traces	0.141 ± 0.001	0.213 ± 0.004	nd	0.106 ± 0.001	0.1056 ± 0.0001	0.107 ± 0.001	nd	0.106 ± 0.001
**18**	0.48 ± 0.02	0.45 ± 0.01	0.517 ± 0.002	0.046 ± 0.007	nd	nd	nd	nd	nd
**19**	0.566 ± 0.004	0.552 ± 0.003	0.536 ± 0.003	0.460 ± 0.001	0.460 ± 0.001	0.460 ± 0.001	0.461 ± 0.001	nd	0.460 ± 0.001
**20**	0.79 ± 0.01	0.67 ± 0.01	0.63 ± 0.01	0.461 ± 0.001	0.462 ± 0.001	0.466 ± 0.001	0.463 ± 0.001	nd	0.463 ± 0.001
**21**	0.475 ± 0.001	0.478 ± 0.001	0.504 ± 0.002	nd	nd	0.461 ± 0.001	nd	nd	nd
**22**	1.25 ± 0.03	1.04 ± 0.02	1.03 ± 0.02	0.462 ± 0.001	nd	0.461 ± 0.001	nd	nd	nd
**23**	1.078 ± 0.02	0.91 ± 0.02	0.911 ± 0.012	0.461 ± 0.001	nd	0.460 ± 0.001	nd	nd	nd
**24**	0.479 ± 0.001	0.48 ± 0.001	0.483 ± 0.001	nd	nd	0.460 ± 0.001	0.464 ± 0.001	0.465 ± 0.001	0.461 ± 0.001
**25**	0.471 ± 0.001	0.474 ± 0.001	0.475 ± 0.001	nd	nd	nd	0.464 ± 0.001	0.467 ± 0.001	0.461 ± 0.001
**26**	nd	0.126 ± 0.003	0.128 ± 0.003	nd	nd	nd	nd	nd	nd
**27**	nd	nd	nd	0.138 ± 0.001	0.43 ± 0.01	0.406 ± 0.001	nd	nd	nd
**28**	nd	nd	nd	0.058 ± 0.001	0.052 ± 0.001	0.066 ± 0.001	nd	nd	nd
**29**	nd	nd	nd	0.07 ± 0.001	0.16 ± 0.003	0.113 ± 0.003	0.108 ± 0.001	0.053 ± 0.001	0.052 ± 0.001
**30**	nd	nd	nd	0.05 ± 0.01	0.126 ± 0.003	0.46 ± 0.02	nd	nd	nd
**31**	nd	nd	nd	0.59 ± 0.02	nd	nd	nd	nd	nd
**32**	nd	nd	nd	0.081 ± 0.001	0.053 ± 0.001	0.16 ± 0.005	nd	nd	nd
**33**	nd	nd	nd	0.460 ± 0.001	0.460 ± 0.001	0.464 ± 0.001	0.462 ± 0.001	nd	0.464 ± 0.001
**34**	nd	nd	nd	nd	0.039 ± 0.004	0.112 ± 0.003	0.067 ± 0.001	0.062 ± 0.001	0.127 ± 0.002
**35**	nd	nd	nd	nd	0.041 ± 0.001	nd	nd	nd	nd
**36**	nd	nd	nd	nd	0.037 ± 0.001	nd	nd	nd	nd
**37**	nd	nd	nd	nd	0.041 ± 0.001	nd	nd	nd	nd
**38**	nd	nd	nd	nd	0.053 ± 0.001	0.088 ± 0.002	nd	nd	nd
**39**	nd	nd	nd	nd	nd	nd	traces	nd	traces
**40**	nd	nd	nd	nd	nd	nd	traces	traces	traces
**41**	nd	nd	nd	nd	nd	nd	traces	traces	traces
**42**	nq	nq	nq	nq	nq	nq	nq	nq	nq
**43**	nq	nq	nq	nq	nq	nq	nq	nq	nq
**44**	nq	nq	nq	nq	nq	nq	nq	nq	nq
TPA	0.78 ± 0.02e	2.55 ± 0.08b	2.66 ± 0.06a	0.153 ± 0.001c	0.100 ± 0.002d	0.31 ± 0.01f	traces	traces	traces
TF	6.47 ± 0.01a	6.28 ± 0.01b	6.3 ± 0.07b	3.55 ± 0.04d	3.13 ± 0.04e	5.28 ± 0.06c	2.708 ± 0.001f	1.146 ± 0.001g	2.709 ± 0.004f
TPC	7.25 ± 0.03c	9.12 ± 0.09a	8.96 ± 0.03b	3.70 ± 0.04e	3.23 ± 0.04f	5.59 ± 0.07d	2.708 ± 0.001g	1.146 ± 0.002h	2.709 ± 0.004g

*Note*: Means in the same line followed by different letters are significantly different according to Tukey's honest significance test (HSD) test (*p* = 0.05). Standard calibration curves: hydroxybenzoic acid (*y* = 208 00*x* + 41 309, *R* ^2^ = 0.9986; LOD = 0.41 µg/mL; LOQ = 1.22 µg/mL; Peak 1); caffeic acid (*y* = 388 345*x* + 406 369; *R* ^2^ = 0.994; LOD = 0.78 µg/mL; LOQ = 1.97 µg/mL; Peaks 2, 3, and 23); *p*‐coumaric acid (*y* = 301 950*x* + 6966.7; *R* ^2^ = 0.999; LOD = 0.68 µg/mL; LOQ = 1.61 µg/mL; Peak 4); (+)‐catechin (*y* = 84 950*x* − 23 200, *R* ^2^ = 1; LOD = 0.17 µg/mL; LOQ = 0.68 µg/mL; Peaks 5, 8, 11, 14, 17, 20, 21, 22, 23, 25, 28, 29, 30, 31, 33, 35, and 36); ferulic acid (*y* = 633 126*x* − 185 462, *R* ^2^ = 0.9990; LOD = 0.20 µg/mL; 1.01 µg/mL; Peaks 6 and 9); chlorogenic acid (*y* = 168 823 − 161 172; *R* ^2^ = 0.9999; LOD = 0.20 µg/mL; LOQ = 0.68 µg/mL; Peaks 7, 12, and 13); protocatechuic acid (*y* = 214 168*x* + 27 102, *R* ^2^ = 0.9999; LOD = 0.14 µg/mL; LOQ = 0.52 µg/mL; Peaks 10 and 21); naringenin (*y* = 18 433*x* + 78 903, *R* ^2^ = 0.9998; LOD = 0.20 µg/mL; LOQ = 0.64 µg/mL; Peak 16); apigenin 6‐*C*‐glucoside (*y* = 107 025*x* − 61 531; *R* ^2^ = 0.9989; LOD = 0.19 µg/mL; LOQ = 0.63 µg/mL; Peak 23); gallic acid (*y* = 131 538*x* + 292 163; *R* ^2^ = 0.9969; LOD = 8.05 µg/mL; LOQ = 24.41 µg/mL; Peak 29); taxifolin (*y* = 39.133*x* − 13.647, *R* ^2^ = 0.02; LOD = 0.07 µg/mL; LOQ = 2.02 µg/mL; Peak 31); quercetin 3‐*O*‐glucoside (*y* = 34 843*x* − 160 173; *R* ^2^ = 0.9998; LOD = 0.21 µg/mL; LOQ = 0.71 µg/mL; Peaks 34, 35, 36, 37, 38, 39, 40, and 41).

Abbreviations: nd, not identified; nq, not quantified; TF, total flavonoids; TPA, total phenolic acids; TPC, total phenolic compounds.

The hydroethanolic extracts, decoction and infusion preparations of *A. squamosa* stem bark, leaves, and seeds were also studied concerning their phenolic composition, and the attained results about the tentative identification of individual compounds were presented in supplementary material in Table SM2. Thirty‐four compounds were detected in the analyzed extracts and preparations, with flavonoids standing out as the major class found.

As in *A. muricata*, procyanidin derivatives prevail in *A. squamosa*, with six compounds (Peaks **6, 9**, **14**, **19**, **22**, and **25**) being tentatively identified by comparison with a (+)‐catechin commercial standard, since procyanidins are made up of simple catechin units. Given the degree of polymerization, the detected compounds were identified as procyanidin dimers (Peaks **9** and **14**), which presented a pseudo‐molecular ion [M−H]^−^ at *m*/*z* 1153 and 577, respectively, trimer (Peak **19**; [M−H]^−^ at *m*/*z* 865), tetramers (Peaks **6** and **25**; [M−H]^−^ at *m*/*z* 1153), and pentamer (Peak **22**; [M−H]^−^ at *m*/*z* 1441), presenting different fragment ions MS^2^ also according to the degree of polymerization. The occurrence of procyanidin derivatives in *A. squamosa* was previously confirmed by Baskaran et al. [47], who were able to identify two procyanidin B2 and a procyanidin trimer, the latter as in our study, this presenting two MS^2^ fragments (451 and 425) like those detected by us. Moreover, other authors [48] found that procyanidin derivatives were also the major compounds found in *A. cherimola* fruits, these being able to identify seven compounds with similar fragmentation patterns as in our study. Following, five catechin derivatives were identified in *A. squamosa* extracts under evaluation, among them dihydroxygallocatechin (Peak **5**; [M−H]^−^ at *m*/*z* 341), epigallocatechin (Peak **16**; [M−H]^−^ at *m*/*z* 305), (−)‐epicatechin hexoside (Peak **15**; [M−H]^−^ at *m*/*z* 431), (−)‐epicatechin (Peak **17**; [M−H]^−^ at *m*/*z* 289), and (+)‐catechin (Peak **11**; [M−H]^−^ at *m*/*z* 289). Manochai et al. [49], have studied the total catechin content of ten *A. squamosa* cultivar peels from Thailand, detecting the presence of these compounds in all of the analyzed samples. However, these authors didn´t perform any individual identification of catechin derivatives. Thus, to the best of our knowledge, our study is the first report regarding the identification of catechin derivatives in different *A. squamosa* plant parts and extracts. Compounds **27**, **28**, **29**, and **33** were tentatively identified as quercetin derivatives by comparison, using quercetin‐3‐*O*‐glucoside as the authentic standard. Quercetin‐3‐*O*‐(6‐*O*‐rhamnosyl)‐galactoside (Peak **27**; [M−H]^−^ at *m*/*z* 609) was identified based on its deprotonated ion and fragmentation pattern. Here, the MS^2^ fragment at *m*/*z* 301 corresponds to the quercetin aglycone, confirming the presence of the quercetin backbone. Following, quercetin‐3‐*O*‐rutinoside (Peak **28**; [M−H]^−^ at *m*/*z* 609), quecetin‐3‐*O*‐glucoside (Peak **29**; [M−H]^−^ at *m*/*z* 463), and quercetin‐3‐*O*‐rhamnoside (Peak **33**; [M−H]^−^ at *m*/*z* 447) were also identified in *A. squamosa* hydroethanolic extracts, infusion and decoction extracts. The fragmentation behavior of the first suggested the loss of a rutinosyl group (−308 Da), allowing its identification. Quercetin‐3‐*O*‐glucoside ([M−H] at *m*/*z* 463), in turn, suggested the detachment of a hexoside unit (−162 Da), while quercetin‐3‐*O*‐rhamnoside ([M−H] at *m*/*z* 447) revealed the loss of rhamnose (−146 Da). Similar quercetin derivatives were previously identified in *A. squamosa* by other authors [50, 51], highlighting their potential biological activities against different health conditions. Kaempferol derivatives were also present, with two individual compounds being identified in *A. squamosa*, namely, kaempferol‐3‐*O*‐(6‐*O*‐rhamnosyl)‐glucoside (Peak **30**) and kaempferol‐3‐*O*‐rutinoside (Peak **31**), both presenting the same deprotonated ion [M−H]^−^ at *m*/*z* 593 and a fragment at *m*/*z* 285. Although these compounds had a similar deprotonated molecular ion, the fragmentation pathway involves the loss of the rutinose disaccharide (−380 u) in a single step, resulting directly in *m*/*z* 285. Although kaempferol derivatives have been previously reported in some *Annona* species [16, 52], only a few studies have reported the occurrence of these compounds in *A. squamosa* [53]. Compounds **26** and **32** ion [M−H]^−^ at *m*/*z* 623, with a unique MS^2^ ion at *m*/*z* 315, according to their elution times, were tentatively identified as isorhamnetin‐3‐*O*‐deoxyhexosyl hexoside and isorhamnetin‐3‐*O*‐rutinoside by comparison with the quercetin 3‐*O*‐glucoside commercial standard, since these share a very similar core structure. The deprotonated ion of the two identified isorhamnetin derivatives reflect their similar sugar composition, and their main differences lie in the fragmentation pattern since the first loses the rhamnose and glucose units sequentially, producing intermediate ions at *m*/*z* 315. In contrast, the second loses the entire rutinose moiety in a single step, forming the *m*/*z* 325 aglycone ion. Like in *A. muricata*, extracts and preparations from *A. squamosa* have presented both naringenin hexoside pentoside (Peak **13**; [M−H]^−^ at *m*/*z* 565 and *m*/*z* 271), chrysin‐6‐*C*‐pentoside‐8‐*C*‐glucoside (Peak **20**; [M−H]^−^ at *m*/*z* 547 and *m*/*z* 253), and eriodictyol‐rutinoside (Peak **24**; [M−H]^−^ at *m*/*z* 595 and *m*/*z* at 287 and 269), these being also first described in the present study.

Concerning phenolic acids, eleven compounds were tentatively identified in *A. squamosa* stem bark, leaves, and seeds hydroethanolic extracts, decoction and infusion preparations, of which caffeic acid derivatives stand out, with four compounds detected (Peaks **2**, **3**, **10**, and **21**). Caffeic acid hexoside (Peak **2**) presented a deprotonated pseudo‐molecular ion [M−H]^−^ at *m*/*z* 341, releasing a fragment ion at *m*/*z* 179, which revealed the loss of a hexose (−162 Da). Dihydrocaffeic acid glucuronide (Peak **3**; [M−H]^−^ at *m*/*z* 391), in turn, presented different MS^2^ ions at *m*/*z* 217, 179, and 135, which correspond to the cleavage of glucuronic acid moiety (−174 Da), followed by the neutral loss of the entire molecule (−176 Da), and its decarboxylation (−44 Da). As for compound **10** ([M−H]^−^ at *m*/*z* 353), the fragmentation pattern allowed its identification as 4‐*O*‐caffeoylquinic acid since deprotonated quinic (*m*/*z* 191) and caffeic (*m*/*z* 179) acid fragments were formed, as well as dehydrated quinic acid (*m*/*z* 173). Finally, methyl‐sulfate caffeic acid hexoside (Peak **21**; [M−H]^−^ at *m*/*z* 437) was also tentatively identified by comparison with authentic standards and the release of two MS^2^ ions, revealing two fragments indicative of the loss of methyl‐sulfate group (−96 Da) and the hexoside moiety (−162 Da). Salman et al. [54] were the first authors to identify caffeic acid in the methanolic extract of *A. squamosa* bark, while Baskaran et al. [47] detected this in the hydroethanolic extract of the fruits pulp. Ferulic acid derivatives in the *A. squamosa* extracts, namely, ferulic acid‐*O*‐hexoside and ferulic acid‐6‐*C*‐hexoside, which presented the same deprotonated ion [M−H]^−^ at *m*/*z* 355, produce a single fragment *m*/*z* 191which correspond to the deprotonated ferulic acid formed by the loss of hexoside moiety (−162 Da). Ferulic acid derivatives were previously identified in *A. squamosa* extracts by other authors [47, 55, 56]. Glucaric acid (Peak **1**; [M−H]− at *m*/*z* 209) was also identified in the studied *A. squamosa* extracts, as well as galloyl‐*p*‐coumaric acid (Peak **3**; [M−H]^−^ at *m*/*z* 391), quinic acid (Peak **8**; [M−H]^−^ at *m*/*z* 191), shikimic acid‐*O*‐hexoside (Peak **18**; [M−H]^−^ at *m*/*z* 337), and 3‐*O*‐ethyl gallic acid sulfate hexoside (Peak **23**; [M−H]^−^ at *m*/*z* 425). Five acetogenin isomers (Peaks **34**–**39**) were also identified, presenting different deprotonated ions [M−H]^−^ and fragmentation patterns. These are primarily derived from plants of the *Annonaceae* species and typically consist of a long aliphatic chain with hydroxyl groups, tetrahydrofuran (THF) rings, and often a lactone group at one end, reason why their fragmentation pattern can be quite complex due to structural diversity. Through the years, different chemical investigations have identified 74 acetogenins in *A. squamosa* [57].

The results attained regarding the quantification of the individual phenolic compounds in the hydroethanolic (Figure S2), decoction and infusion preparations of *A. squamosa* stem bark, leaves, and seeds are presented in Table 2. Among the studied plant parts, the leaves presented the highest concentration of total phenolic compounds in all the analyzed extracts (10.9, 9.5, and 6.66 mg/g extract, respectively, as above). Of these, epigallocatechin (Peak **16**) was detected in both extracts at high concentrations (2.582, 1.11, and 0.59 mg/g extract, respectively, for hydroethanolic extracts, decoction, and infusion preparations), being only overcome by (−)‐epicatechin (Peak **17**) in the leaves hydroethanolic extract, here representing the major compound detected (2.999 mg/g extract). Epigallocatechin was also spotted in the stem bark extracts at lower amounts, while (−)‐epicatechin was only detected in leaves. Quercetin‐3‐*O*‐rutinoside (Peak **28**) was likely noticed in high concentrations in extracts and preparations of *A. squamosa* leaves, with concentrations ranging from 1.655 to 2.109 mg/g extract, the hydroethanolic one presenting the highest amount. In the infusion preparations, other compounds appear in good amounts, such as procyanidin tetramer (Peak **25**; 0.554 mg/g extract) and quercetin‐3‐*O*‐ramnoside (Peak **33**; 0.541 mg/g extract). Regarding *A. squamosa* stem bark, epigallocatechin, isorhamnetin‐3‐*O*‐deoxyhesoxyl hexoside (Peak **26**), and quercetin‐3‐*O*‐(6‐*O*‐rhamnosyl)‐galactosidase (Peak **27**) stands out in the hydroethanolic extract, while in decoction and infusion preparations also good amounts of quercetin‐3‐*O*‐rutinoside and kaempferol‐3‐*O*‐(6‐*O*‐rhamnosyl)‐glucoside was noticed, varying from 0.460 to 0.491 mg/g extract. Overall, seeds from *A. squamosa* presented the lowest amount of total phenolic compounds, namely, de hydroethanolic extract (0.282 mg/g extract), followed by decoction (1.146 mg/g extract) and infusion (1.36 mg/g extract) preparations. In these, the major contribution was performed by procyanidin dimer in the hydroethanolic extract, and quercetin‐3‐*O*‐(6‐*O*‐rhamnosyl)‐galactosidase and quercetin‐3‐*O*‐rutinoside in decoction and infusion preparations. Kumar et al. [58] evaluated the phenolic profile of 30 *A. squamosa* genotypes leaf extracts, finding much lower total phenolic concentrations (212.8–1478.4 µg/g extract) since only 7 compounds were identified in these extracts. To the best of our knowledge, this is the first report on the individual compounds noticed in different extracts of *A. squamosa* plant parts, with previous studies only focusing on the total phenolic content.

**TABLE 2:** Content (mg/g extract) of the phenolic compounds identified in the leaves, stem bark, and seeds of *Annona squamosa* hydroethanolic extracts, decoction, and infusion preparations (mean ± SD, *n* = 3).

	Leaves			Stem bark			Seeds		
Peaks	Hydroethanolic	Decoction	Infusion	Hydroethanolic	Decoction	Infusion	Hydroethanolic	Decoction	Infusion
**1**	0.134 ± 0.004	0.802 ± 0.026	0.294 ± 0.01	0.33 ± 0.001	0.129 ± 0.004	0.365 ± 0.008	0.055 ± 0.001	0.019 ± 0.001	0.034 ± 0.001
**2**	nd	nd	traces	traces	traces	traces	nd	nd	nd
**3**	traces	traces	traces	traces	traces	traces	traces	nd	nd
**4**	0.016 ± 0.001	0.019 ± 0.001	traces	0.035 ± 0.001	traces	0.039 ± 0.001	0.02 ± 0.001	traces	0.006 ± 0.001
**5**	0.044 ± 0.001	0.054 ± 0.001	0.037 ± 0.001	0.057 ± 0.001	0.034 ± 0.001	0.088 ± 0.002	0.039 ± 0.001	0.035 ± 0.001	0.044 ± 0.001
**6**	0.267 ± 0.002	nd	0.224 ± 0.006	nd	nd	nd	0.052 ± 0.001	0.074 ± 0.001	0.108 ± 0.002
**7**	0.047 ± 0.001	0.142 ± 0.004	0.106 ± 0.001	nd	nd	nd	nd	nd	nd
**8**	0.112 ± 0.001	0.439 ± 0.008	0.248 ± 0.005	nd	nd	nd	nd	nd	nd
**9**	nd	nd	nd	nd	0.07 ± 0.001	nd	nd	nd	nd
**10**	0.14 ± 0.001	0.114 ± 0.001	0.111 ± 0.001	nd	nd	nd	nd	nd	nd
**11**	nd	nd	nd	0.326 ± 0.004	nd	0.205 ± 0.005	nd	nd	nd
**12**	0.058 ± 0.001	0.059 ± 0.001	0.039 ± 0.001	nd	nd	nd	nd	nd	nd
**13**	traces	nd	traces	nd	nd	nd	nd	nd	nd
**14**	nd	0.738 ± 0.006	0.499 ± 0.002	0.109 ± 0.003	0.037 ± 0.001	0.239 ± 0.006	0.064 ± 0.001	0.040 ± 0.001	0.048 ± 0.001
**15**	0.243 ± 0.008	nd	nd	nd	nd	nd	nd	nd	nd
**16**	2.582 ± 0.009	1.11 ± 0.031	0.59 ± 0.009	0.575 ± 0.009	0.149 ± 0.004	0.06 ± 0.001	nd	nd	nd
**17**	2.999 ± 0.002	0.823 ± 0.009	0.409 ± 0.004	nd	nd	nd	nd	nd	nd
**18**	0.051 ± 0.001	0.163 ± 0.003	nd	nd	nd	nd	nd	nd	nd
**19**	nd	0.572 ± 0.008	nd	nd	0.030 ± 0.001	nd	0.053 ± 0.001	0.055 ± 0.001	0.055 ± 0.001
**20**	0.351 ± 0.004	0.562 ± 0.002	0.375 ± 0.002	traces	traces	0.025 ± 0.002	traces	traces	0.139 ± 0.002
**21**	nd	nd	nd	nd	nd	nd	traces	traces	traces
**22**	nd	nd	nd	0.041 ± 0.001	0.141 ± 0.004	0.522 ± 0.002	nd	nd	nd
**23**	nd	nd	nd	nd	nd	nd	traces	traces	traces
**24**	nd	nd	nd	0.124 ± 0.001	0.121 ± 0.001	0.187 ± 0.002	nd	nd	nd
**25**	0.254 ± 0.008	0.372 ± 0.001	0.554 ± 0.006	nd	nd	nd	nd	nd	nd
**26**	nd	nd	nd	0.559 ± 0.003	0.461 ± 0.001	0.467 ± 0.001	nd	nd	nd
**27**	nd	nd	nd	0.502 ± 0.001	0.460 ± 0.001	0.466 ± 0.001	nd	0.460 ± 0.001	0.460 ± 0.001
**28**	2.109 ± 0.058	1.924 ± 0.013	1.655 ± 0.042	0.471 ± 0.001	0.4651 ± 0.001	0.491 ± 0.001	nd	0.462 ± 0.001	0.462 ± 0.001
**29**	0.512 ± 0.001	0.506 ± 0.001	0.508 ± 0.002	0.472 ± 0.001	nd	0.466 ± 0.001	nd	nd	nd
**30**	nd	nd	nd	0.485 ± 0.001	0.461 ± 0.001	0.469 ± 0.001	nd	nd	nd
**31**	0.473 ± 0.001	0.486 ± 0.001	0.473 ± 0.001	0.483 ± 0.001	nd	0.469 ± 0.001	nd	nd	nd
**32**	nd	nd	nd	0.462 ± 0.001	nd	nd	nd	nd	nd
**33**	0.55 ± 0.003	0.571 ± 0.003	0.541 ± 0.003	nd	nd	nd	nd	nd	nd
**34**	nd	nd	nd	nd	nd	nd	nq	nq	nq
**35**	nd	nd	nd	nd	nd	nd	nq	nq	nq
**36**	nd	nd	nd	nd	nd	nd	nq	nq	nq
**37**	nd	nd	nd	nd	nd	nd	nq	nq	nq
**38**	nd	nd	nd	nd	nd	nd	nq	nq	nq
**39**	nd	nd	nd	nd	nd	nd	nq	nq	nq
TPA	0.60 ± 0.01c	1.79 ± 0.04a	0.84 ± 0.02b	0.42 ± 0.01e	0.163 ± 0.004f	0.49 ± 0.01d	0.113 ± 0.001g	0.055 ± 0.001i	0.083 ± 0.001h
TF	10.3 ± 0.3a	7.7 ± 0.1b	5.8 ± 0.1c	4.61 ± 0.03d	2.39 ± 0.01f	4.07 ± 0.03e	0.168 ± 0.003g	1.091 ± 0.001i	1.27 ± 0.01h
TPC	10.9 ± 0.3a	9.5 ± 0.2b	6.66 ± 0.01c	5.03 ± 0.05d	2.56 ± 0.02f	4.56 ± 0.04e	0.282 ± 0.004i	1.146 ± 0.003h	1.36 ± 0.01g

*Note*: Means in the same line followed by different letters are significantly different according to Tukey's honest significance (HSD) test (*p* = 0.05). Standard calibration curves: hydroxybenzoic acid (*y* = 208 00*x* + 41 309, *R* ^2^ = 0.9986; LOD = 0.41 µg/mL; LOQ = 1.22 µg/mL; Peak 1); caffeic acid (*y* = 388 345*x* + 406 369; *R* ^2^ = 0.994; LOD = 0.78 µg/mL; LOQ = 1.97 µg/mL; Peaks 2, 3, and 18); *p*‐coumaric acid (*y* = 301 950*x* + 6966.7; *R* ^2^ = 0.999; LOD = 0.68 µg/mL; LOQ = 1.61 µg/mL; Peak 4); (+)‐catechin (*y* = 84 950*x* − 23 200, *R* ^2^ = 1; LOD = 0.17 µg/mL; LOQ = 0.68 µg/mL; Peaks 5, 6, 9, 10, 13, 14, 15, 16, 18, 21, and 24); ferulic acid (*y* = 633 126*x* − 185 462, *R* ^2^ = 0.9990; LOD = 0.20 µg/mL; 1.01 µg/mL; Peaks 7 and 12); chlorogenic acid (*y* = 168 823 − 161 172; *R* ^2^ = 0.9999; LOD = 0.20 µg/mL; LOQ = 0.68 µg/mL; Peak 14); protocatechuic acid (*y* = 214 168*x* + 27 102, *R* ^2^ = 0.9999; LOD = 0.14 µg/mL; LOQ = 0.52 µg/mL; Peaks 8 and 18); naringenin (*y* = 18 433*x* + 78 903, *R* ^2^ = 0.9998; LOD = 0.20 µg/mL; LOQ = 0.64 µg/mL; Peak 16); apigenin 6‐*C*‐glucoside (*y* = 107 025 − 61 531; *R* ^2^ = 0.9989; LOD = 0.19 µg/mL; LOQ = 0.63 µg/mL; Peak 23); gallic acid (*y* = 131 538*x* + 292 163, *R* ^2^ = 0.9969, LOD = 8.05 µg/mL and LOQ = 24.41 µg/mL; Peak 26); taxifolin (*y* = 39.133*x* − 13.647, *R* ^2^ = 0.02; LOD = 0.07 µg/mL; LOQ = 2.02 µg/mL; Peak 30); quercetin 3‐*O*‐glucoside (*y* = 34 843*x* − 160 173; *R* ^2^ = 0.9998; LOD = 0.21 µg/mL; LOQ = 0.71 µg/mL; Peaks 32, 33, 34, and 35).

Abbreviations: nd, not identified; nq, not quantified; TF, total flavonoids; TPA, total phenolic acids; TPC, total phenolic compounds.

The tentative identification of individual phenolic compounds in *A. senegalensis* stem bark, leaves, and seeds hydroethanolic extracts, decoction and infusion preparations are described in supplementary material in Table SM3, in which data regarding the retention time, *λ*_max_, pseudo‐molecular ions, and major fragment ions in MS^2^ are also presented. Thirty‐seven compounds were identified in this species, of which twenty‐nine flavonoids and eight phenolic acids. Among flavonoids, and as in the previous *Annona* species described, procyanidins with different polymerization degrees prevail, with 13 compounds identified as dimers (Peaks **5**, **9**, **15**, and **16**; [M−H]^−^ at *m*/*z* 577), trimers (Peaks **19**, **23**, **24**, and **28**; [M−H]^−^ at *m*/*z* 865), tetramers (Peaks **7**, **21**, and **27**; [M−H]^−^ at *m*/*z* 1153), and pentamers (Peaks **22** and **25**; [M−H]^−^ at *m*/*z* 1441). Procyanidin dimers and trimers isomers reveal the same fragmentation pattern, which corresponds to the loss of a catechol group (*m*/*z* at 451; −126 Da), retro‐Diels–Alder fragmentation (*m*/*z* at 425; −152 Da), rearrangements (*m*/*z* 407; −170 Da), and the deprotonated ion of either catechin or epicatechin (*m*/*z* at 289) [59]. Procyanidin tetramers and pentamers, in turn, produce main fragment ions coincident with the loss of two or three monomers, respectively (*m*/*z* 1153, 865, and 577; −288 Da each), and a final fragment at *m*/*z* 289 (monomeric unit) [59]. As far as we can infer, this is the first report of procyanidins in *A. senegalensis*, this being although previously identified in other plants of the same genus, as before mentioned. Quercetin derivatives come next, with five compounds being identified. Quercetin‐3‐*O*‐(6‐*O*‐rhamnosyl)‐galactoside (Peak **30**) and quercetin‐3‐*O*‐rutinoside (Peak **31**) presented the same deprotonated ion [M−H]^−^ at *m*/*z* 609 and a MS^2^ ion at *m*/*z* 301, indicative of the loss of a rutinose (−308 Da) in both compounds. Despite quercetin‐3‐*O*‐glucoside (Peak **32**), quercetin‐3‐*O*‐pentoside (Peak **35**) and quercetin‐3‐*O*‐rhamnoside (Peak **37**) presented the same fragment of the before described compound, this presented a deprotonated ion [M−H]^−^ at *m*/*z* 463, and 447, respectively, revealing the loss of glucose, a pentose, and rhamnose monomers. Very few investigations have reported the occurrence of quercetin derivatives in *A. senegalensis* [60, 61] since this seems to be a less studied species. Our investigation also allowed the tentative identification of catechin derivatives in *A. senegalensis* extracts and preparations. Dihydroxygallocatechin (Peak **4**; [M−H]^−^ at *m*/*z* 341) presented a fragmentation pattern concordant with the loss of H_2_O molecules (−36 Da) and a fragment at *m*/*z* 169 that resulted from retro‐Diels–Alder cleavage of the C‐ring. (−)‐Catechin and (−)‐epicatechin (Peaks **12** and **18**; [M−H]^−^ at 289), in turn, produce a single fragment at *m*/*z* 245, revealing a minor fragment due to the loss of hydroxyl groups. Finally, epigallocatechin (Peak **17**; [M−H]^−^ at 305) presented main MS^2^ ions representative of retro‐Diels–Alder cleavages (*m*/*z* 179 and 125). Vildina and colleagues [60] also identified catechin derivatives in *A. senegalensis* leaf extracts; however, this is the only report of these type of compounds found in the literature. Two isorhamnetin derivatives were spotted in the analyzed *A. senegalensis* extracts, specifically isorhamnetin‐3‐*O*‐deoxyhexosyl hexoside (Peak **29**) and isorhamnetin‐3‐*O*‐rutinoside, presenting the same deprotonated ion [M−H]^−^ at *m*/*z* 623 and a MS^2^ ion at *m*/*z* 315, revealing the loss of rutinose (−308 Da) in both compounds. Likewise, kaempferol‐3‐*O*‐(6‐*O*‐rhamnosyl)‐glucoside (Peak **33**) and kaempferol‐3‐*O*‐rutinoside (Peak **34**) presented the same [M−H]^−^ at *m*/*z* 593 and a single fragment at *m*/*z* 285, also concordant with the loss of rutinose. In the present investigation, both kaempferol and isorhamnetin derivatives were first described in *A. senegalensis*. As in the other *Annona* species in study, naringenin hexoside pentoside (Peak **13**), chrysin‐6‐*C*‐pentoside‐8‐*C*‐glucoside (Peak **20**), and eriodictyol‐rutinoside (Peak **26**) were identified in *A. senegalensis* extract by comparison with commercial standards.

Eight phenolic acids were tentatively identified in *A. senegalensis* extracts, of which caffeic acid derivatives stand out with three compounds detected (Peaks **2**, **11**, and **14**). Dihydroxycaffeic acid glucoronide appears first, presenting a deprotonated ion [M−H]^−^ at *m*/*z* 391 and daughter fragments at *m*/*z* 217, 179, and 135 that points to a loss of glucuronic acid, CO_2_, and further breakdown of the fragment, respectively. Compounds **11** and **14,** were positively identified as caffeoylquinic acids given their deprotonated ion [M−H]^−^ at *m*/*z* 353 and ion fragments of quinic acid (*m*/*z* 191) and decarboxylated caffeic acid (*m*/*z* 179). Once again, this is the first report regarding the occurrence of caffeic acid derivatives in *A. senegalensis* hydroethanolic extracts, decoction and infusion preparations. Other phenolic acids were concomitantly identified in this studied, namely, glucaric (Peak **1**; [M−H]^−^ at *m*/*z* 209), galloyl‐*p*‐coumaric (Peak **3**; [M−H]^−^ at *m*/*z* 315), ferulic (Peak **6**; [M−H]^−^ at *m*/*z* 355), quinic (Peak **8**; [M−H]^−^ at *m*/*z* 191), and *p*‐coumaryolquinic (Peak **10**; [M−H]^−^ at *m*/*z* 337), as in the other studied *A. muricata* and *A. squamosa* hydroethanolic, decoction and infusion preparation.

Data about the quantification of the individual compounds identified in *A. senegalensis* stem bark, leaves, and seeds hydroethanolic extracts (Figure S3), decoction and infusion preparations are presented in Table 3. Among the analyzed *A. senegalensis* plant parts, leaves stand out, presenting the higher concentration in total phenolic compounds in both hydroethanolic extracts (22.2 mg/g extract), decoction (8.7 mg/g extract), and infusion (10.6 mg/g extract) preparations. In this plant structure the major compound identified was quercetin‐3‐*O*‐rutinoside, mainly in the hydroethanolic extract (3.417 mg/g extract), being also prevalent in leaves decoction (2.386 mg/g extract), and infusion (2.39 mg/g extract) preparations. (−)‐Epicatechin appears next in *A. senegalensis* leaves hydroethanolic extract (2.616 mg/g extract), although being present in much lower amounts in the remaining decoction and infusion preparations (0.261 and 0.501 mg/g extract). In these latter, and besides the above mentioned major compound identified, quercetin‐3‐*O*‐(6‐*O*‐rhamnosyl)‐glucoside (1.05 mg/g extract, each), and isorhamnetin‐3‐*O*‐rutinoside (0.712 mg/g extract, each) were also present in good concentrations, the latter being the major compound found in *A. senegalensis* stem barks, with values ranging from 0.728 to 0.906 mg/g extract. In this plant structure, quercetin‐3‐*O*‐rutinoside was also present in satisfactory concentrations in all of the studied extracts (from 0.594 to 0.665 mg/g extract), followed by also good amounts of isorhamnetin‐3‐*O*‐deoxyhexosyl hexoside and quercetin‐3‐*O*‐glucoside, only detected in decoction and infusion preparations, reason why these sums the higher amount of total phenolic compounds. Finally, in *A. senegalensis* seed extract, several compounds were found in very similar amounts, namely, isorhamnetin‐3‐*O*‐deoxyhexosyl hexoside, quercetin‐3‐*O*‐rutinoside, quercetin‐3‐*O*‐glucoside, and isorhamnetin‐3‐*O*‐rutinoside, with values ranging from 0.461 to 0.482 mg/g extract.

**TABLE 3:** Content (mg/g extract) of the phenolic compounds identified in the leaves, stem bark, and seeds of *Annona senegalensis* hydroethanolic extracts, decoction, and infusion preparations (mean ± SD, *n* = 3).

	Leaves			Stem bark			Seeds		
Peaks	Hydroethanolic	Decoction	Infusion	Hydroethanolic	Decoction	Infusion	Hydroethanolic	Decoction	Infusion
**1**	0.202 ± 0.005	0.178 ± 0.005	nd	0.209 ± 0.005	0.155 ± 0.001	0.159 ± 0.002	0.035 ± 0.001	0.139 ± 0.005	0.049 ± 0.001
**2**	nd	traces	nd	traces	traces	traces	traces	traces	traces
**3**	0.042 ± 0.004	0.16 ± 0.003	0.115 ± 0.004	0.03 ± 0.001	0.013 ± 0.001	0.015 ± 0.001	0.008 ± 0.001	traces	0.024 ± 0.001
**4**	nd	0.068 ± 0.001	nd	0.056 ± 0.001	0.048 ± 0.001	0.049 ± 0.001	0.035 ± 0.001	0.039 ± 0.001	0.043 ± 0.002
**5**	0.849 ± 0.006	nd	0.43 ± 0.009	nd	nd	nd	nd	nd	nd
**6**	nd	0.103 ± 0.002	0.106 ± 0.002	nd	nd	nd	nd	nd	nd
**7**	0.491 ± 0.001	nd	nd	0.110 ± 0.002	0.078 ± 0.001	0.101 ± 0.001	0.129 ± 0.004	0.081 ± 0.002	0.332 ± 0.001
**8**	nd	0.12 ± 0.003	nd	nd	nd	nd	nd	nd	nd
**9**	0.465 ± 0.001	nd	0.313 ± 0.008	0.083 ± 0.001	nd	nd	nd	nd	nd
**10**	nd	0.132 ± 0.001	nd	nd	nd	nd	nd	nd	nd
**11**	nd	0.155 ± 0.001	nd	nd	nd	nd	nd	nd	nd
**12**	0.254 ± 0.007	0.254 ± 0.008	0.577 ± 0.006	0.100 ± 0.002	0.053 ± 0.001	nd	0.046 ± 0.001	0.112 ± 0.003	0.153 ± 0.004
**13**	nd	traces	nd	nd	nd	nd	nd	nd	nd
**14**	0.194 ± 0.002	nd	0.137 ± 0.002	nd	nd	nd	nd	nd	nd
**15**	0.504 ± 0.005	nd	0.279 ± 0.003	0.088 ± 0.001	nd	nd	nd	nd	nd
**16**	1.56 ± 0.001	nd	0.361 ± 0.003	0.218 ± 0.004	0.092 ± 0.008	0.124 ± 0.001	0.066 ± 0.001	0.192 ± 0.005	0.166 ± 0.005
**17**	0.791 ± 0.009	nd	nd	0.141 ± 0.003	0.09 ± 0.004	nd	0.051 ± 0.001	0.185 ± 0.004	0.119 ± 0.003
**18**	2.616 ± 0.004	0.261 ± 0.003	0.501 ± 0.001	nd	0.069 ± 0.002	nd	0.051 ± 0.001	0.115 ± 0.003	0.334 ± 0.001
**19**	1.586 ± 0.006	nd	nd	0.164 ± 0.003	0.184 ± 0.001	0.142 ± 0.001	0.134 ± 0.004	0.095 ± 0.002	0.437 ± 0.002
**20**	nd	0.123 ± 0.001	nd	nd	nd	nd	nd	nd	nd
**+**	1.88 ± 0.005	nd	0.261 ± 0.003	0.11 ± 0.001	nd	nd	nd	nd	nd
**22**	0.676 ± 0.001	nd	0.255 ± 0.001	nd	0.059 ± 0.001	0.066 ± 0.001	0.065 ± 0.001	0.07 ± 0.001	0.112 ± 0.003
**23**	0.426 ± 0.004	nd	nd	nd	0.094 ± 0.001	0.082 ± 0.002	0.071 ± 0.001	0.034 ± 0.001	0.187 ± 0.006
**24**	0.262 ± 0.005	nd	0.329 ± 0.002	nd	nd	nd	nd	nd	nd
**25**	0.31 ± 0.007	nd	nd	nd	nd	nd	nd	nd	nd
**26**	nd	0.228 ± 0.001	nd	0.149 ± 0.001	0.163 ± 0.001	0.155 ± 0.002	0.112 ± 0.001	0.119 ± 0.001	0.124 ± 0.001
**27**	0.25 ± 0.006	0.301 ± 0.001	0.301 ± 0.001	nd	nd	nd	nd	nd	nd
**28**	0.547 ± 0.009	nd	nd	nd	nd	nd	nd	nd	nd
**29**	nd	nd	nd	nd	0.484 ± 0.001	0.489 ± 0.001	0.461 ± 0.001	0.468 ± 0.001	0.463 ± 0.001
**30**	0.949 ± 0.008	1.05 ± 0.003	1.05 ± 0.013	nd	nd	nd	nd	nd	nd
**31**	3.417 ± 0.102	2.386 ± 0.007	2.39 ± 0.07	0.665 ± 0.004	0.623 ± 0.001	0.594 ± 0.004	0.469 ± 0.001	0.463 ± 0.001	0.482 ± 0.001
**32**	0.531 ± 0.001	0.478 ± 0.001	0.478 ± 0.001	nd	0.487 ± 0.001	0.469 ± 0.001	0.461 ± 0.001	0.465 ± 0.001	0.472 ± 0.001
**33**	0.510 ± 0.001	0.485 ± 0.001	0.485 ± 0.001	nd	nd	nd	nd	0.495 ± 0.001	nd
**34**	0.523 ± 0.001	0.494 ± 0.001	0.494 ± 0.001	nd	nd	nd	nd	nd	nd
**35**	0.643 ± 0.003	0.521 ± 0.002	0.521 ± 0.002	nd	nd	nd	nd	nd	nd
**36**	0.563 ± 0.003	0.502 ± 0.001	0.502 ± 0.001	0.906 ± 0.007	0.815 ± 0.004	0.728 ± 0.002	0.465 ± 0.001	0.475 ± 0.001	0.477 ± 0.001
**37**	1.157 ± 0.001	0.712 ± 0.008	0.712 ± 0.008	nd	nd	nd	nd	nd	nd
TPA	0.44 ± 0.01b	0.92 ± 0.02a	0.36 ± 0.01c	0.29 ± 0.01d	0.216 ± 0.001f	0.222 ± 0.003e	0.077 ± 0.001i	0.18 ± 0.02g,h	0.116 ± 0.003h
TF	21.8 ± 0.4a	7.8 ± 0.1c	10.2 ± 0.1b	2.73 ± 0.03g	3.29 ± 0.02f	2.95 ± 0.01	2.58 ± 0.01h	3.37 ± 0.02e	3.9 ± 0.1d
TPC	22.2 ± 0.5a	8.7 ± 0.1c	10.6 ± 0.1b	3.03 ± 0.04g	3.51 ± 0.02d,e	3.17 ± 0.02f	2.66 ± 0.02h	3.55 ± 0.03d	10.2 ± 0.1b

*Note*: Standard calibration curves: hydroxybenzoic acid (*y* = 20 800*x* + 41 309; *R* ^2^ = 0.9986; LOD = 0.41 µg/mL; LOQ = 1.22 µg/mL; Peak 1); quercetin 3‐*O*‐glucoside (*y* = 34 843*x* − 160 173; *R* ^2^ = 0.9998; LOD = 0.21 µg/mL; LOQ = 0.71 µg/mL; Peaks 2, 28, 29, 30, 31, 32, 33, 34, 35, 36, 37); *p*‐coumaric acid (*y* = 301 950*x* + 6966.7; *R* ^2^ = 0.999; LOD = 0.68 µg/mL; LOQ = 1.61 µg/mL; Peak 3); (+)‐catechin (*y* = 84 950*x* − 23 200, *R* ^2^ = 1; LOD = 0.17 µg/mL; LOQ = 0.68 µg/mL; Peaks 4, 5, 7, 9, 12, 15, 16, 17, 18, 19, 21, 22, 23, 24, 25, 27, 28); ferulic acid (*y* = 633 126*x* − 185 462, *R* ^2^ = 0.9990; LOD = 0.20 µg/mL; 1.01 µg/mL; Peak 6); chlorogenic acid (*y* = 168 823 − 161 172; *R* ^2^ = 0.9999; LOD = 0.20 µg/mL; LOQ = 0.68 µg/mL; Peak 10, 11, 14); hydroxybenzoic acid (*y* = 20 800*x* + 41 309, *R* ^2^ = 0.9986; LOD = 0.41 µg/mL; LOQ = 1.22 µg/mL; Peak 8); naringenin (*y* = 18 433*x* + 78 903, *R* ^2^ = 0.9998; LOD = 0.20 µg/mL; LOQ = 0.64 µg/mL; Peak 13); apigenin 6‐*C*‐glucoside (*y* = 107 025 − 61 531; *R* ^2^ = 0.9989; LOD = 0.19 µg/mL; LOQ = 0.63 µg/mL; Peak 20); taxifolin (*y* = 39.133*x* − 13.647, *R* ^2^ = 0.02; LOD = 0.07 µg/mL; LOQ = 2.02 µg/mL; Peak 33).

Abbreviations: nd, not identified; nq, not quantified; TF, total flavonoids; TPA, total phenolic acids; TPC, total phenolic compounds.

Overall, in *A. muricata*, *A. squamosa*, and *A. senegalensis* leaves, stem bark, and seed hydroethanolic extracts, decoction, and infusion preparations, flavonoids stand out as the major class of compounds identified. Moreover, the leaves of all of the studied *Annona* species displayed the highest concentration in total phenolic compounds, followed by stem barks, and seeds.

### 2.2 Antioxidant and Antimalarial Activities

The results obtained regarding the antioxidant activity of the studied *A. muricata*, *A. senegalensis*, and *A. squamosa* leaves, stem bark, and seeds hydroethanolic extracts, decoction, and infusion preparations are presented in Table 4. Overall, the leaves of all *Annona* species investigated presented higher antioxidant activity, followed by the stem barks and seeds. Besides that, the hydroethanolic extracts of both leaves and stem barks of these species perform a better antioxidant capacity, presenting lower EC_50_ values than those of the remaining extracts. Three different assays were used to evaluate the antioxidant capacity of the extracts since these measures different parameters linked to the bioactivity. Regarding *A. muricata*, the greater antioxidant capacity was observed in leaves hydroethanolic extract (EC_50_ = 10.2 µg/mL) using the DPPH assay. Despite that, in this structure, also very good results were attained in the reducing power assay performed in the remaining extracts, with EC_50_ values ranging from 15.3 to 36 µg/mL. In *A. muricata* stem bark, on the other hand, the best antioxidant performance was accomplished with the reducing power assay and mainly in the infusion preparations (EC_50_ = 15.1 µg/mL), with also good results being accomplished in the DPPH (from 29.0 to 84 µg/mL). In the first, the accomplished EC_50_ values were lower than those of trolox for the same assay (EC_50_ = 43 ± 2 µg/mL. As for *A. muricata* seeds, a higher antioxidant capacity was also accomplished using the reducing power assay all of the extracts, in which infusion stands out (EC_50_ = 103 µg/mL). Regarding the TBARS assay, the EC_50_ values were much higher than those of the other assays and the trolox (EC_50_ = 21.8 ± 0.2 µg/mL. The antioxidant capacity of *A. muricata* extracts was previously investigated by other authors [26, 62, 63], who also reported a higher bioactive potential positively correlated with the phenolic composition of its extracts.

**TABLE 4:** Antioxidant (EC_50_, µg/mL) and antimalarial (EC_50_, µg/mL) activities of leaves, stem bark, and seeds of the *Annona muricata*, *Annona squamosa*, and *Annona senegalensis* hydroethanolic extracts, decoction, and infusion preparations (mean ± SD; *n* = 3).

	Leaves			Stem bark			Seeds		
	Hydroethanolic	Decoction	Infusion	Hydroethanolic	Decoction	Infusion	Hydroethanolic	Decoction	Infusion
*A. muricata*									
Antioxidant activity^a^									
DPPH	47 ± 2e	10.2 ± 0.7i	22 ± 2h	33 ± 2f	84 ± 6d	29.0 ± 0.4g	145 ± 8b	273 ± 7a	138 ± 11c
Reducing Power	36 ± 2d	15.3 ± 0.4 h,i	30.1 ± 0.9e	15.5 ± 0.1 g,h	16.0 ± 0.7f	15.1 ± 0.1i	108 ± 6b	111 ± 6a	103 ± 15c
TBARS	119 ± 10f	108 ± 10i	113 ± 2g	129 ± 7d,e	130 ± 11d	110 ± 3h	136 ± 13b	193 ± 14a	132 ± 9c
Antimalarial activity^b^, ^*^									
Pf3D7	21 ± 6	> 100	> 100	> 50	> 50	> 50	5 ± 1	> 100	> 100
*A. squamosa*									
Antioxidant activity^a^									
DPPH	9.86 ± 0.02i	23 ± 2g	26.9 ± 0.9f	12.6 ± 0.2h	45 ± 1e	57 ± 1c	204 ± 13a	187 ± 4b	47 ± 1d
Reducing powe	16.0 ± 0.9i	17.9 ± 0.5h	25 ± 2g	65 ± 2f	105 ± 5e	125 ± 3d	152 ± 7a	144 ± 8b	128 ± 5c
TBARS	83 ± 5h	115 ± 12e	126 ± 5d	102 ± 30g	115 ± 32e	200 ± 24b	188 ± 8c	209 ± 11a	109 ± 3f
Antimalarial activity^b^, ^*^									
Pf3D7	> 50	> 100	> 100	> 100	> 100	> 100	24 ± 1	> 50	41 ± 13
*A. senegalensis*									
Antioxidant activity^a^									
DPPH	12.9 ± 0.1h	27 ± 1d	19.5 ± 0.4e	18 ± 1f	11.9 ± 0.02i	14.4 ± 0.6g	57 ± 3a	47 ± 4b	37.3 ± 0.1c
Reducing power	12.1 ± 0.3i	17.3 ± 0.9e	13.74 ± 0.02h	19.3 ± 0.4d	14.75 ± 0.04g	15.2 ± 0.2f,g	75 ± 3a	59 ± 2b	51 ± 3c
TBARS	85 ± 10c	119 ± 17a	104 ± 3b	29 ± 1h	15.9 ± 0.2i	20 ± 5g	63 ± 2d	59 ± 1e	40 ± 1f
Antimalarial activity^b^									
Pf3D7	5.0 ± 0.4c	> 50	> 50	38.6b	> 100	> 100	49.3a	> 100	> 100

*Note*: Means in the same line followed by different letters are significantly different according to Tukey's honest significance (HSD) test (*p* = 0.05). Positive controls—Trolox EC_50_ values: 43 ± 2 µg/mL (DPPH), 30 ± 1 µg/mL (reducing power), and 21.8 ± 0.2 µg/mL (TBARS); ellipticine GI_50_ values: 1.4 ± 0.1 µg/mL; chloroquine EC_50_ values: 0.010 ± 0.002 µg/mL.

^a^ EC_50_: extract concentration corresponding to 50% antioxidant activity or 0.5 absorbance for the reducing power assay;

^b^ EC_50_: half maximal inhibitory concentration on *Plasmodium falciparum* 3D7 strain.

* Significant differences (*p* < 0.001) between the two samples were assessed by a Student's *t*‐test.

As for *A. squamosa*, the hydroethanolic extract of the leaves displayed the best antioxidant capacity in all of the performed assays, mainly in DPPH (EC_50_ = 9.86 µg/mL). Despite that, the other extracts also presented good antioxidant assets, with EC_50_ values ranging from 16.0 and 126 µg/mL, the higher values being attributed to the TBARS assay. In *A. squamosa* stem barks, in turn, the DPPH assay was more efficient than the others, with the hydroethanolic extract presenting the lower EC_50_ among the studied extracts and antioxidant assays. Regarding seeds, like *A. muricata*, the infusion preparation performed the best antioxidant capacity, which is more pronounced in the DPPH assay. Mustanir et al. [64] evaluated the antioxidant activity of *A. squamosa* leaf methanolic extract using the DPPH assay. Their results showed that the ethanolic extract performed a higher antioxidant capacity than the remaining, presenting IC_50_ values of 6.67 ppm. In this study, the authors attributed the high bioactive potential of the extract to the presence of phytochemical compounds, among them 4,4′‐((*p*‐phenylene)diisopropylidene) diphenol, dodecanoic acid, methyl ester, and phthalic acid, isobutyl 2‐methylpent‐3‐yl ester.

Finally, among the studied *Annona* species, *A. senegalensis* performed the higher antioxidant activity, associated with the higher concentration of total phenolic compounds in this species (Table 4). Regarding *A. senegalensis* leaves, the best results were achieved using the reducing power assay in all extracts, presenting EC_50_ values ranging from 12.1 to 17.3 µg/mL, lower than that those of the Trolox (EC_50_ = 30 ± 1 µg/mL. Stem barks, in turn, also offered a great antioxidant capacity, similar to that of the leaves, ranging from 11.9 to 29 µg/mL, with also good results being accomplished in the TBARS assay. Although with a lower antioxidant performance, the seeds from *A. senegalensis* also presented good results in this field. Diallo et al. [65] recently studied the phytochemical profile and the antioxidant capacity of *A. senegalensis* leaves and stem bark hydroethanolic extracts. Their results showed that both extracts performed similar antioxidant activities presenting high inhibition percentages, namely, 95.06% for the leaves and 92.87% for the bark, highlighting its correlation with bioactive compounds in both plant parts.

The antimalarial activity of the studied *Annona* plant parts is presented in Table 4. Regarding *A. muricata*, only the hydroethanolic extract of the leaves (EC_50_ = 21 µg/mL) and seeds (EC_50_ = 5 µg/mL) presented some antimalarial effects, while in *A. squamosa* only the seeds were able to perform it, name the hydroethanolic extract (EC_50_ = 24 µg/mL) and infusion preparation (EC_50_ = 41 µg/mL). As for *A. senegalensis*, some antimalarial activity was observed in all of the studied hydroethanolic extracts of the different plant parts, this being much more pronounced in the leaves (EC_50_ = 15 µg/mL), followed by the stem bark (EC_50_ = 38.6 µg/mL), and the seeds (EC_50_ = 49.3 µg/mL). Several studies have reported the antimalarial activity of the extracts of different parts of *Annona* species. Onohuean et al. [66] studied the *A. muricata* leaves ethanolic extracts' capacity in reduce *Plasmodium berghei* parasitemia in BALB/c mice. This author reported that at the tested doses (100, 200, and 400 mg/kg), the extract was able to attenuate the *P. berghei*‐induced elevation of parasitemia and cytokines (TNF‐α, IL‐5, and IL‐6) in vivo during the experimental period, though not as much as chloroquine, used as a positive control. Sawant et al. [67], in turn, investigated the antimalarial potential of the seeds of *A. squamosa*, finding that the oily fraction, Annomaal, demonstrated pronounced antimalarial activity with low IC_50_ (1.25 µg/mL) against *Plasmodium falciparum* in vitro. Moreover, these authors also reported that crude seed extract and Annomaal significantly inhibited the growth of *P. berghei* parasites in vivo with 58.47% and 61.11% chemo suppression, respectively. In our study, only the hydroethanolic and infusion extracts of *A. squamosa* presented some antimalarial activity, per the reported study. To validate the antimalarial effectiveness of *A. senegalensis* methanolic crude extracts, Ngbolua et al. [68] used two malaria parasite strains (*P. falciparum* FcM29 and *Plasmodium yoelii* subsp. *nigeriensis*), reporting a moderate in vitro and a weak in vivo antiplasmodial activity. These results may be due to the extract solvent used (methanol) since, in our study, only the hydroethanolic extract of the different *A. senegalensis* plant parts were able to demonstrate some antimalarial activity.

### 2.3 Antimicrobial Activity

The results of the antimicrobial activity of *A. muricata*, *A. senegalensis*, and *A. squamosa* leaves, stem bark, and seeds hydroethanolic extracts, decoction, and infusion preparations are presented in Table 5. Regarding *A. muricata*, the higher antibacterial potential was performed by the seed's infusion preparations against *Bacillus cereus* (MIC = 0.6 mg/mL), while some bioactivity was also observed by the stem bark hydroethanolic extract against the same strain (MIC = 2.5 mg/mL). *Yersinia enterocolitica* was substantially inhibited by both leaves and stem bark decoction preparations of *A. muricata* (MIC = 1.25 and 2.5 mg/mL, respectively), as well as *Staphylococcus aureus*, which was inhibited by the hydroethanolic extracts (MIC = 2.5 mg/mL) and infusion preparation (MIC = 1.25 mg/mL) stem bark. As for *A. squamosa*, both the studied extracts of the plant stem bark displayed the higher antimicrobial capacity against *S. aureus*, presenting MIC values of 1.25 mg/mL. Moreover, leaf hydroethanolic extracts effectively inhibited the growth of *B. cereus* and *S. aureus* (MIC = 2.5 mg/mL). In *A. squamosa* seeds, no antimicrobial activity was observed against the utilized bacterial strains. The results attained for *A. senegalensis* reveal that this species was much more effective in inhibiting the growth of a higher number of bacterial strains, with the best capacity being exhibited by the leaves hydroethanolic extract against *S. aureus* and by all the studied stem bark extracts against *B. cereus* (MIC = 0.6 mg/mL). These latter also showed a good antibacterial capacity against *S. aureus* (MIC = 1.25 mg/mL), *Salmonella enterica* (MIC = 2.5 mg/mL; in infusion and decoction preparations), *Escherichia coli* (MIC = 2.5 mg/mL; in decoction preparations), and *Enterobacter cloacae* (MIC = 2.5 mg/mL; hydroethanolic extract). In *A. senegalensis* leaves, the studied extracts were also effective in the inhibition of other strains, namely, *B. cereus* (MIC = 1.25 mg/mL) by the hydroethanolic extract, as well as the decocted preparation against *S. aureus*, *E. coli*, and *Pseudomonas aeruginosa* and the hydroethanolic extract against *S. enterica* (MIC = 2.5 mg/mL). Finally, regarding seeds, the decocted preparations were able to inhibit a more significant number of bacterial strains, namely, *E. cloacae* (MIC = 1.25), *E. coli*, *B cereus*, and *Listeria monocytogenes* (MIC = 2.5 mg/mL), while its hydroethanolic extract only significantly inhibited the growth of *Y. enterocolitica* (MIC = 1.25 mg/mL). Here, once again, the higher antimicrobial potential of *A. senegalensis* may be linked to its high content in phenolic compounds. Regarding the antifungal activity, only infusions preparations of *A. muricata* and *A. squamosa* inhibited the growth of *Aspergillus fumigatus* (MIC = 2.5 mg/mL), while *Aspergillus brasiliensis* was inhibited by the decoction preparation of *A. senegalensis* (MIC = 2.5 mg/mL). Several studies have confirmed the antimicrobial capacity of the *Annona* species in different plant parts and extracts [69, 70, 71, 72], which gives space to the use of these plants in the development of various types of nutraceuticals, cosmetics, and other products.

**TABLE 5:** Antimicrobial activity of leaves, stem bark, and seeds of the *Annona muricata*, *Annona squamosa*, and *Annona senegalensis* hydroethanolic extracts, decoction, and infusion preparations (mg/mL).

		*A. muricata*			*A. squamosa*			*A. senegalensis*					
		Leaves	Stem bark	Seeds	Leaves	Stem bark	Seeds	Leaves	Stem bark	Seeds	Positive controls		
		MIC/MBC	MIC/MBC	MIC/MBC	MIC/MBC	MIC/MBC	MIC/MBC	MIC/MBC	MIC/MBC	MIC/MBC	MIC/MBC	MIC/MBC	MIC/MBC
Antibacterial activity											Streptomicin	Methicilin	Ampicillin
*Enterobacter cloacae*	Hydroethanolic	> 10/> 10	> 10/> 10	> 10/> 10	> 10/> 10	> 10/> 10	> 10/> 10	**5/**> 10	**2.5/**> 10	> 10/> 10	0.007/0.007	nt/nt	0.15/0.15
	Decoction	> 10/> 10	> 10/> 10	> 10/> 10	> 10/> 10	> 10/> 10	> 10/> 10	10/> 10	5/> 10	1.25/> 10	0.007/0.007	nt/nt	0.15/0.15
	Infusion	> 10/> 10	> 10/> 10	> 10/> 10	> 10/> 10	> 10/> 10	> 10/> 10	10/> 10	5/> 10	5/> 10	0.007/0.007	nt/nt	0.15/0.15
*Escherichia coli*	Hydroethanolic	10/> 10	> 10/> 10	> 10/> 10	10/> 10	10/> 10	> 10/> 10	5/> 10	5/> 10	10/> 10	0.01/0.01	nt/nt	0.15/0.15
	Decoction	10/> 10	> 10/> 10	> 10/> 10	10/> 10	10/> 10	> 10/> 10	2.5/> 10	2.5/> 10	2.5/> 10	0.01/0.01	nt/nt	0.15/0.15
	Infusion	10/> 10	> 10/> 10	> 10/> 10	> 10/> 10	10/> 10	> 10/> 10	10/> 10	5/> 10	5/> 10	0.01/0.01	nt/nt	0.15/0.15
*Pseudomonas aeruginosa*	Hydroethanolic	10/> 10	> 10/> 10	> 10/> 10	10/> 10	10/> 10	> 10/> 10	5/> 10	> 10/> 10	> 10/> 10	0.06/0.06	nt/nt	0.63/0.63
	Decoction	> 10/> 10	> 10/> 10	10/> 10	> 10/> 10	> 10/> 10	> 10/> 10	2.5/> 10	> 10/> 10	> 10/> 10	0.06/0.06	nt/nt	0.63/0.63
	Infusion	5/> 10	> 10/> 10	> 10/> 10	10/> 10	> 10/> 10	> 10/> 10	10/> 10	> 10/> 10	> 10/> 10	0.06/0.06	nt/nt	0.63/0.63
*Salmonella enterica*	Hydroethanolic	10/> 10	10/> 10	> 10/> 10	10/> 10	> 10/> 10	> 10/> 10	2.5> 10	5/> 10	10/> 10	0.007/0.007	nt/nt	0.15/0.15
	Decoction	> 10/> 10	10/> 10	> 10/> 10	10/> 10	> 10/> 10	> 10/> 10	10/> 10	2.5/> 10	> 10/> 10	0.007/0.007	nt/nt	0.15/0.15
	Infusion	> 10/> 10	> 10/> 10	> 10/> 10	> 10/> 10	> 10/> 10	> 10/> 10	10/> 10	2.5/> 10	> 10/> 10	0.007/0.007	nt/nt	0.15/0.15
*Yersinia enterocolitica*	Hydroethanolic	10/10	5/> 10	> 10/> 10	10/10	> 10/> 10	> 10/> 10	5/10	> 10/> 10	1.25/> 10	0.007/0.007	nt/nt	0.15/0.15
	Decoction	1.25/> 10	2.5/2.5	> 10/> 10	10/10	10/> 10	> 10/> 10	5/> 10	10/> 10	10/> 10	0.007/0.007	nt/nt	0.15/0.15
	Infusion	> 10/> 10	10/> 10	> 10/> 10	10/10	> 10/> 10	> 10/> 10	5/5	10/> 10	> 10/> 10	0.007/0.007	nt/nt	0.15/0.15
*Bacillus cereus*	Hydroethanolic	10/> 10	2.5/> 10	> 10/> 10	2.5/> 10	10/> 10	> 10/> 10	1.25/> 10	0.6/> 10	5/> 10	0.007/0.007	nt/nt	nt/nt
	Decoction	> 10/> 10	> 10/> 10	> 10/> 10	> 10/> 10	10/> 10	> 10/> 10	10/> 10	0.6/> 10	2.5/> 10	0.007/0.007	nt/nt	nt/nt
	Infusion	> 10/> 10	10/> 10	0.6/> 10	10/> 10	10/> 10	> 10/> 10	10/> 10	0.6/> 10	10/> 10	0.007/0.007	nt/nt	nt/nt
*Listeria monocytogenes*	Hydroethanolic	10/> 10	> 10/> 10	> 10/> 10	10/> 10	> 10/> 10	> 10/> 10	> 10/> 10	10/> 10	5/> 10	0.007/0.007	nt/nt	0.15/0.15
	Decoction	> 10/> 10	> 10/> 10	> 10/> 10	> 10/> 10	> 10/> 10	> 10/> 10	> 10/> 10	10/> 10	2.5/> 10	0.007/0.007	nt/nt	0.15/0.15
	Infusion	> 10/> 10	> 10/> 10	> 10/> 10	> 10/> 10	> 10/> 10	> 10/> 10	10/> 10	10/> 10	5/> 10	0.007/0.007	nt/nt	0.15/0.15
*Staphylococcus aureus*	Hydroethanolic	10/> 10	2.5/> 10	> 10/> 10	2.5/> 10	1.25/> 10	> 10/> 10	0.6/> 10	1.25/> 10	10/> 10	0.007/0.007	0.007/0.007	0.15/0.15
	Decoction	> 10/> 10	1.25/> 10	> 10/> 10	> 10/> 10	1.25/> 10	10/> 10	2.5/> 10	1.25/> 10	10/> 10	0.007/0.007	0.007/0.007	0.15/0.15
	Infusion	> 10/> 10	> 10/> 10	> 10/> 10	10/> 10	1.25/> 10	> 10/> 10	10/> 10	1.25/> 10	> 10/> 10	0.007/0.007	0.007/0.007	0.15/0.15
Antifungal activity											Ketoconazole		
*Aspergillus brasiliensis*	Hydroethanolic	> 10/> 10	> 10/> 10	> 10/> 10	> 10/> 10	> 10/> 10	5/> 10	> 10/> 10	> 10/> 10	5/> 10	0.06/0.125		
	Decoction	> 10/> 10	> 10/> 10	10/> 10	> 10/> 10	10/> 10	10/> 10	> 10/> 10	2.5/> 10	5/> 10			
	Infusion	> 10/> 10	> 10/> 10	10/> 10	> 10/> 10	10/> 10	> 10/> 10	> 10/> 10	> 10/> 10	5/> 10			
*Aspergillus fumigatus*	Hydroethanolic	**10/**> 10	**5/**> 10	> 10/> 10	**10/**> 10	**10/**> 10	> 10/> 10	**10/**> 10	**10/**> 10	**10/**> 10	0.5/1		
	Decoction	**10/**> 10	**10/**> 10	> 10/> 10	**10/**> 10	**10/**> 10	**10/**> 10	**10/**> 10	**10/**> 10	**10/**> 10			
	Infusion	**10/**> 10	**10/**> 10	**2.5/**> 10	**10/**> 10	**10/**> 10	**2.5/**> 10	**5/**> 10	**10/**> 10	**10/**> 10			

Abbreviations: MBC, minimal bactericidal concentration; MFC, minimum fungal concentrations; MIC, minimal inhibitory concentration; nt, not tested.

Despite the promising findings, this study has several limitations that should be considered. The composition of plant extracts can vary depending on environmental conditions, harvesting time, and plant part used, which may affect the reproducibility and consistency of bioactive properties [73]. In addition, the results were obtained using in vitro assays, which do not fully replicate the complexity of in vivo biological systems. Therefore, further studies, including in vivo experiments and clinical evaluations, are necessary to confirm the therapeutic potential of these extracts.

## 3 Conclusion

The studied *Annona* species revealed distinct and diverse polyphenolic profiles, mainly composed of flavonoids, phenolic acids, and acetogenins. Among them, *A. muricata* exhibited the highest diversity of phenolic compounds, including epigallocatechin, quercetin derivatives, catechins, acetogenins, and gallic acid. *A. squamosa* was characterized by elevated levels of epigallocatechin, quercetin and caffeic acids, while *A. senegalensis* showed procyanidin, epicatechin, quercetin as predominant compounds. These species‐specific phenolics contribute to the differentiation of each *Annona* species and may underline their reported antioxidant and therapeutic properties. *A. senegalensis* extracts demonstrated the strongest antioxidant activity, while *A. muricata* seed hydroethanolic extract showed superior antimalarial effects. Antimicrobial activities varied among species and extracts, with notable effects against specific bacterial and fungal strains.

This study provided further knowledge about *A. muricata*, *A. squamosa*, and *A. senegalensis* from Angola. However, the antioxidant and antimicrobial results reported in this study should be considered as preliminary in vitro indicators of bioactivity. The observed MIC and minimum MBC values, particularly when higher than 10 mg/mL, indicate weak antimicrobial activity. Therefore, these findings cannot justify or validate traditional therapeutic uses without further, more comprehensive pharmacological and toxicological investigations. Such additional studies are essential to confirm efficacy, safety, and potential mechanisms of action under physiological conditions.

## 4 Experimental Section

### 4.1 Standard and Reagents

Trolox (6‐hydroxy‐2,5,7,8‐tetramethylchroman‐2‐carboxylic acid) was purchased from Sigma (St. Louis, MO, USA). Formic and acetic acids were purchased from Prolabo (VWR International, France). Ethyl acetate (99.8%) was from Fisher Scientic (Lisbon, Portugal). Phenolic compound standards were purchased from Extrasynthese (Genay, France). Fetal bovine serum (FBS), l‐glutamine, Hank's balanced salt solution (HBSS), trypsin–EDTA (ethyl‐enediaminetetraacetic acid) were from Hyclone (Logan, Utah, USA). Acetic acid, ellipticine, sulforhodamine B (SRB), dimethyl sulfoxide (DMSO), trypan blue, tri‐chloroacetic acid (TCA) and tris (tris(hydroxymethyl)amino‐methane) were from Sigma Chemical Co. (St. Louis, MO, USA). Microorganisms, Mueller–Hinton (MH) agar, malt agar (MA) as well as positive controls (ampicillin, streptomycin, methicillin, and ketoconazole) were purchase from Frilabo, Porto, Portugal. All other chemicals and solvents were of analytical grade and purchased from common sources. Water was treated in a Milli‐Q water purification system (TGI Pure Water Systems, Greenville, SC, USA).

### 4.2 Sampling and samples preparation

**FIGURE 1:**
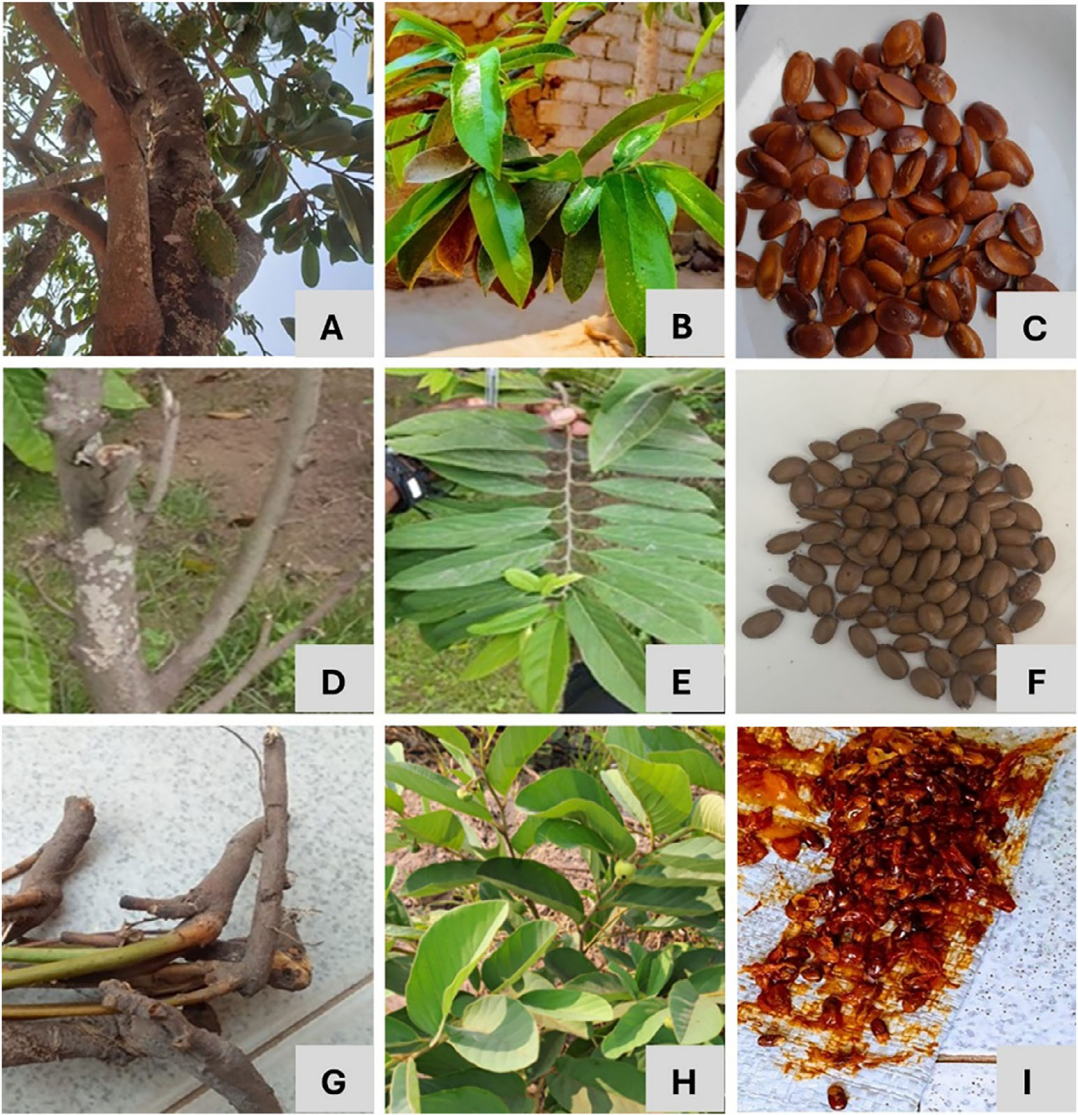
Stem bark, leaves, and seeds from *A. muricata* (a–c, respectively), *A. squamosa* (d–f, respectively), and *A. senegalensis* (g–i, respectively). Photos by Josefa Rangel.

*A. muricata*, *A. squamosa*, and *A. senegalensis* seeds, stem bark, and leaves (Figure 1) were collected during fieldwork conducted in Angola in January 2022. *A. muricata* and *A. senegalensis* were collected near the Cangandala National Park, Malanje Province (9°37′20.49″ S/16°24′29.39″ E). *A. squamosa* was collected in Sumbe, Cuanza Sul (11°16′40.92″ S/13°53′55.02″ E). Plant specimens were identified by the first and last authors, and vouchers [*A. muricata*: J. Rangel 87 (LISC131240); *A. senegalensis*: J. Rangel 88 (LISC131241); and *A. squamosa*: J. Rangel 89 (LISC131242)] were deposited at the LISC Herbarium, University of Lisbon, Portugal. After botanical identification, samples were cleaned to remove foreign materials, freeze‐dried using a FreeZone 4.5 lyophilizer, kept in a dry environment at room temperature, and protected from light. Freeze‐dried samples were ground and stored in the dark until further analysis.

### 4.3 Hydroethanolic Extracts, Decoctions, and Infusions Preparations

The *A. muricata*, *A. squamosa*, and *A. senegalensis* stem bark, leaves, and seeds samples were prepared in hydroethanolic extracts, decoction and infusion preparations to evaluate their composition in phenolic compounds and the in vitro bioactive properties.

Hydroethanolic extracts were prepared given a previously described procedure [74]. In brief, 1 g of each lyophilized sample was twice suspended in 30 mL of ethanol/water (80:20; v/v), stirred at 150 rpm for 1 h at room temperature, and then filtered through Whatman no. 4 paper. Following, the ethanol was removed by evaporation, using a rotary evaporator (Büchi R‐210, Flawil, Switzerland) at 40°C, and the aqueous phase frozen and lyophilized (FreeZone 4.5 model 7750031, Labconco, Kansas City, MO, USA) for further analysis.

Decoctions preparations, each sample (3 g) was boiled with 100 mL of distilled water for 5 min on a heating plate and then filtered through Whatman filter paper no. 4. The obtained extracts were frozen and lyophilized to obtain a dried extract and stored until further analysis [75].

For infusions, preparations were made by adding 100 mL of boiled distilled water to each sample (2 g), allowing it to rest for 5 min, and then filtering through Whatman filter paper no. 4. The optimized sample quantities (1 g for hydroethanolic extraction and 3 g for decoction and infusion) were before determined to be ideal for each method, maximizing phenolic compound extraction, ensuring that the extracts accurately represent the plant's phenolic profile. The resulting extracts were then frozen and lyophilized [76].

### 4.4 HPLC–DAD–ESI/MS*^n^* Analysis of Phenolic Compounds

The phenolic composition of the stem bark, leaves, and stems of *A. muricata*, *A. squamosa*, and *A. senegalensis* was analyzed using hydroethanolic extracts, decoctions, and infusions. The hydroethanolic extracts were redissolved in an ethanol:water mixture (80:20, v/v), while the decoctions and infusions were redissolved in water. All preparations were adjusted to a final concentration of 10 mg/mL for the analysis and subsequently filtered using 0.22 µm disposable filter disks. The analysis was performed as previously described by Bessada et al. [74] in a high‐performance liquid chromatography (HPLC) system (Dionex Ultimate 3000 UPLC, Thermo Scientific, San Jose, CA, USA) coupled with a diode‐array detector (DAD), using 280 and 370 nm as preferred wavelengths, and a Linear Ion Trap (LTQ XL) mass spectrometer (MS, Thermo Finnigan, San Jose, CA, USA) equipped with an electrospray ionization (ESI) source. Separation was accomplished in a Waters Spherisorb S3 ODS‐2 C18 column (3 µm, 4.6 mm × 150 mm; Waters, Milford, MA, USA). The identification of each individual compound was made using the Xcalibur 4.6 software (Thermo Fisher Scientific), based on the chromatographic data obtained (retention time, UV–Vis spectra and mass), and compared with existing standards or earlier defined data in the literature. Quantification of each compound was accomplished using a seven‐level calibration curves built on the UV signal of available standard compounds. When precise standards were not available, calibration curves of the most comparable standards were used. Results are expressed in mg/g extract.

### 4.5 Bioactive properties

#### 4.5.1 Antioxidant activity

For the antioxidant assays, the sample preparation involved different solvents for each type of extract. The lyophilized hydroethanolic extracts were re‐dissolved in an ethanol: water mixture (80:20, v/v), while the decoction and infusion preparations were re‐dissolved in water. Following this initial re‐dissolution, all samples, regardless of their original preparation method, underwent a series of successive dilutions, ranging from 10 mg/mL down to 0.0025 mg/mL. For DPPH (2,2‐diphenyl‐1‐picrylhydrazyl) radical‐scavenging activity, each dilution (30 µL) was mixed with 270 µL of a methanolic solution containing a concentration of 6 × 10^−5^ mol/mL of DPPH radicals. The DPPH radical‐scavenging activity was calculated measuring the absorbance at 515 nm in a microplate reader (ELX800 Biotek microplate reader; Bio‐Tek Instruments Inc., Winooski, VT, USA) and recorded as the percentage of DPPH discoloration by comparing the absorbance at 515 nm using the equation: [(*A*_DPPH_ − AS)/*A*_DPPH_] × 100, where ADPPH is the absorbance of the DPPH solution and AS is the absorbance of the solution when the sample extract has been added at a particular level [77].

Reducing power was performed using the Microplate Reader described above. The different concentrations of the extracts (0.5 mL) were mixed with sodium phosphate buffer (200 mmol/L, pH 6.6, 0.5 mL) and potassium ferricyanide (1% w/v, 0.5 mL). For each concentration, the mixture was incubated at 50°C for 20 min, and trichloroacetic acid (10% w/v, 0.5 mL) was added. The mixture (0.8 mL) was poured in the 48‐wells, alongside deionized water (0.8 mL) and ferric chloride (0.1% w/v, 0.16 mL), and the absorbance was measured at 690 nm [77]. For the EC_50_ calculation, the following equation was used: (ARP − AS), where ARP corresponds to the absorbance of the control and AS is the absorbance measured for the solution when the sample extract has been added at a particular level. The EC_50_ value was determined as the concentration providing 50% of the maximum reducing power.

The capacity of the extracts to inhibit the formation of thiobarbituric acid reactive substances (TBARS) was assessed in *Sus scrofa* brain homogenates as an oxidizable substrate. Here, the lipid peroxidation capacity was calculated by the decrease in TBARS, evaluated by the color intensity of malondialdehyde–thiobarbituric acid (MDA–TBA) measured at 532 nm. The inhibition ratio was calculated using the formula: [(*A* − *B*)/*A*] × 100%, where *A* and *B* were the absorbance of the control and the sample solutions, respectively. The results were expressed in EC_50_ values, which corresponds to the sample concentration providing 50% of antioxidant activity or 0.5 absorbance for the reducing power assay. Trolox was used as the positive control [77].

#### 4.5.2 Antimalarial Activity

*P. falciparum* 3D7 strain was cultured using established protocols at hematocrit of 4% in RPMI‐1640 medium, supplemented with 0.05 mg/mL gentamicin and 5% Albumax II. The parasites were kept in an environment with 5% carbon dioxide, at 37°C, and synchronized using 5% D‐sorbitol. The drug efficacy of different compounds was evaluated using a SYBR green fluorescence‐based assay [78]. In brief, parasites in the ring stage (> 80%) were set at a parasitemia of 0.5% and 2% hematocrit, then incubated in 96‐well plates with various concentrations of the test compounds, in duplicate. After 72 h of incubation, the plates underwent freeze‐thaw cycles, and 100 µL of the resulting lysate was transferred to fresh black 96‐well plates containing 100 µL of lysis buffer (composed of 20 mM Tris, 5 mM EDTA, 0.008% wt/v saponin, 0.08% v/v Triton X‐100, and 0.4 µL/mL of 10× SYBR Green I). The plates were then left in the dark for 60 min before fluorescence was measured using the CLARIOstar system (Labtech) at an excitation wavelength of 490 nm and an emission wavelength of 540 nm. Parasite inhibition percentages were calculated by comparing the growth in treated wells to untreated controls, and EC_50_ values (µg/mL) were determined through nonlinear regression analysis in Prism GraphPad software.

#### 4.5.3 Antimicrobial Activity

In this assay, bacteria frequently associated with foodborne diseases were used, namely, Gram‐negative *E. cloacae* (ATCC 49741), *E. coli* (ATCC 25922), *P. aeruginosa* (ATCC 9027), *S. enterica* (ATCC 13076), and *Y. enterocolitica* (ATCC 8610), and Gram‐positive *B. cereus* (ATCC 11778), *L. monocytogenes* (ATCC 19111), and *S. aureus* (ATCC 25923). All these microorganisms are purchase at Frilabo. The bacteria were incubated at 37°C an appropriate fresh medium, for 24 h before analysis to maintain the exponential growth phase.

The antibacterial activity of the hydroethanolic extracts, decoction and infusion preparations was determined using the broth microdilution method to the rapid *p*‐iodonitrotetrazolium chloride (INT) colorimetric assay [79]. The samples were first of all dissolved in 5% (v/v) DMSO and 95% of autoclaved distilled water to give a final concentration of 20 mg/mL for the stock solution. 90 µL of this concentration was added in the first well (96‐well microplate) in duplicate with 100 µL of tryptic soy broth (TSB). In the remaining wells 90 µL of TSB medium were added. Then the samples were serially diluted to obtain the concentration ranges (10–0.03125 mg/mL). To finish, 10 µL of inoculum (standardized at 1.5 × 10^6^ colony forming unit [CFU]/mL) was added at all the well assuring the presence of 1.5 × 10^5^ CFU. Two negative controls were prepared, one with TSB and another one with the extract. Two positive controls were prepared with TSB and each inoculum and another with medium, antibiotics, and bacteria. Ampicillin was used as a positive control for all bacterial strains, streptomycin was used as a positive control for all bacterial strains except for *B. cereus*, and methicillin for *S. aureus* only. The microplates were incubated at 37°C for 24 h. The MIC of samples was detected following addition (40 µL) of 0.2 mg/mL *p*‐iodonitrotetrazolium chloride (INT) and incubation at 37°C for 30 min. MIC was defined as the lowest concentration that inhibits the visible bacterial growth determinate by change the coloration from yellow to pink if the microorganisms are viable. For the determination of MBC, 10 µL of liquid from each well that showed no change in color was plated on solid medium, blood agar (7% sheep blood) and incubated at 37°C for 24 h. The lowest concentration that yielded no growth determines the MBC. MBC was defined as the lowest concentration required to kill bacteria. The results were expressed as minimum inhibitory concentration (MIC, mg/mL) and minimum bactericidal concentration (MBC, mg/mL).

#### 4.5.4 Antifungal Activity

The antifungal activity was performed according to described by the authors [80]. *A. fumigatus* (ATCC 204305) and *A. brasiliensis* (ATCC 16404) were used. The organisms were obtained from Frilabo. The micromycetes were maintained on MA and the cultures stored at 4°C and were further placed in new medium and incubated at 25°C for 72 h. To investigate the antifungal activity, the fungal spores were washed from the surface of agar plates with sterile 0.85% saline containing 0.1% Tween 80 (v/v). The spore suspension was adjusted with sterile saline to a concentration of approximately 1.0 × 10^5^ in a final volume of 100 µL per well. The samples were first of all dissolved in 5% (v/v) DMSO and 95% of autoclaved distilled water to give a final concentration of 10 mg/mL for the stock solution. Afterward, 90 µL of this concentration was added in the first well (96‐well microplate) in duplicate with 100 µL of malt extract broth (MEB). In the remaining wells 90 µL of medium MEB were placed. Then the samples were serially diluted obtaining the concentration ranges (10–0.03125 mg/mL). MIC determinations were performed by a serial dilution technique using 96‐well microplate. The lowest concentrations without visible growth (at the binocular microscope) were defined as MICs. The fungicidal concentration (MFC) was determined by serial subcultivation of a 2 µL of tested compounds dissolved in medium and inoculated for 72 h, into microplates containing 100 µL of MEB per well and further incubation 72 h at 26°C. The lowest concentration with no visible growth was defined as MFC indicating 99.5% killing of the original inoculum. Commercial fungicide ketoconazole (Frilabo), was used as positive control.

### 4.6 Statistical Analysis

The results for the phenolic composition and bioactive properties are presented as mean ± SD (*n* = 3). The statistical analysis was performed by using the SPSS v. 23.0 software for Windows (IBM Corp., Armonk, NY, USA) and using the one‐way analysis of variance (ANOVA). Post hoc comparisons of means were carried out using Tukey's HSD test (*p* < 0.05) when statistically significant differences were detected. Some results of the antimalarial activity were assessed using Student's *t*‐test to determine significant differences between two samples, with *α* = 0.05.

## Author Contributions

**Josefa Rangel**: formal analysis, investigation, writing – original draft. **Ângela Liberal**: formal analysis, investigation, writing – original draft. **Tiane C. Finimundy**: formal analysis, investigation, methodologies, writing – review and editing. **Tânia S. P. Pires**: formal analysis. **Pedro Cravo**: formal analysis, methodologies. **Maria Conceição Silva**: formal analysis, methodologies. **Gustavo Cassiano**: formal analysis, methodologies. **Lillian Barros**: methodologies, visualization, funding acquisition. **Maria M. Romeiras**: conceptualization, visualization, supervision, writing – review and editing, **Ângela Fernandes**: methodologies, conceptualization, visualization, supervision, writing – original draft.

## Conflicts of Interest

The authors declare no conflicts of interest.

## Supporting information




**Supporting file 1**: Supplementary data to this article is provided as supplementary files attached to the manuscript, including **Tables SM1–SM3** and **Figures S1–S3**.

## Data Availability

The data that support the findings of this study are available in the Supporting Information material of this article.
