# Supplementary material for: Phenolic Profile and Bioactive Prospects of Wild Annona Species From Angola
Source: Chem Biodivers. 2025 Dec 10;23(2):e03294. doi: 10.1002/cbdv.202503294 (PMC12860525; doi:10.1002/cbdv.202503294)
Supplement: Supplementary file 1 — Supporting file 1: Supplementary data to this article is provided as supplementary files attached to the manuscript, including Tables SM1–SM3 and Figures S1–S3. [file CBDV-23-e03294-s001.docx]

**SUPPLEMENTARY DATA**

**Phenolic profile and bioactive prospects of wild *Annona* species from Angola**

Josefa Rangel^1,2,3†^, Ângela Liberal^3,†^, Tiane C. Finimundy^3^, Tânia S. P. Pires^3^, Pedro Cravo^4^, Maria Conceição Silva^4^, Gustavo Capatti Cassiano^4^, Lillian Barros^3,*^, Maria M. Romeiras^1,5,*^, Ângela Fernandes^3^

^1^Linking Landscape, Environment, Agriculture and Food Research Center (LEAF), Associated Laboratory TERRA, Instituto Superior de Agronomia (ISA), Universidade de Lisboa, Tapada da Ajuda, 1340-017 Lisboa, Portugal

^2^Centro de Botânica, Universidade Agostinho Neto, Avenida Ho Chi Minh, Prédio do CNIC, 1◦ andar, ala esquerda, Luanda, Angola

^3^CIMO, LA SusTEC, Instituto Politécnico de Bragança, Campus de Santa Apolónia, 5300-253 Bragança, Portugal

^4^Global Global Health and Tropical Medicine (GHTM), Associate Laboratory in Translation and Innovation Towards Global Health, (LA-REAL), Instituto de Higiene e Medicina Tropical, (IHMT), Universidade NOVA de Lisboa, (UNL), 1349-008 Lisboa, Portugal.

^5^ Centre for Ecology, Evolution and Environmental Changes (cE3c) & CHANGE - Global Change and Sustainability Institute, Faculdade de Ciências, Universidade de Lisboa, Campo Grande, 1749-016 Lisboa, Portugal

† These authors contributed equally to this work

Corresponding authors: L. Barros ([lillian@ipb.pt](mailto:lillian@ipb.pt)) and M. Romeiras ([mmromeiras@isa.ulisboa.pt](mailto:mmromeiras@isa.ulisboa.pt))

**Table SM1 ⏐** Phenolic compounds tentatively identified in the leaves, stem bark, and seeds of *A. muricata* hydroethanolic extracts, decoction and infusion preparations. The retention time (Rt), wavelength of maximum absorption (λmax) in the UV-Vis region, and mass spectral data are presented.

| **Peaks** | **Rt (min)** | **λmax (nm)** | **[M-H]^-^ *m/z*** | **MS^2^ (*m/z*)** | **Tentative Identification** |
| --- | --- | --- | --- | --- | --- |
| **1** | 4.31 | 324 | 209 | 191(100) | Glucaric acid |
| **2** | 4.52 | 276 | 341 | 179(100) | Caffeic acid-hexoside |
| **3** | 4.69 | 321 | 391 | 217(100),179(55),135(8) | Dihydrocaffeic acid-glucoronide |
| **4** | 5.13 | 312 | 315 | 163(100) | galloyl-*p*-coumaric acid |
| **5** | 5.43 | 313 | 341 | 305(100),169(23) | Dihydroxygallocatechin |
| **6** | 5.69 | 311 | 355 | 193(100) | Ferulic acid-hexoside |
| **7** | 5.73 | 312 | 675 | 337(100),191(28) | 3,4-di-*p*-coumaroylquinic acid |
| **8** | 5.77 | 281 | 1153 | 865(22), 713(4), 577(33), 575(16), 561(20), 289(100) | Procyanidin tetramer isomer I |
| **9** | 6.05 | 313 | 355 | 191(100) | Ferulic acid-*O*-hexoside |
| **10** | 6.13 | 305 | 191 | 173(100),127(12) | Quinic acid |
| **11** | 6.41 | 281 | 577 | 451(38),425(67),407(100),289(76) | Procyanidin dimer isomer I |
| **12** | 6.44 | 325 | 337 | 163(100),119(11) | *p*-coumaroylquinic acid |
| **13** | 6.73 | 322 | 353 | 191(32),179(52)173(100) | 4-*O*-caffeoylquinic acid |
| **14** | 7.24 | 288 | 289 | 245(100) | (+)-Catechin |
| **15** | 7.28 | 312 | 355 | 191(100) | Ferulic acid-6-*C-*hexoside |
| **16** | 7.34 | 308 | 565 | 271(100) | Naringenin-hexoside-pentoside |
| **17** | 8.02 | 288 | 577 | 451(38),425(67),407(100),289(76) | Procyanidin dimer isomer II |
| **18** | 8.72 | 295 | 431 | 289(100) | (-)-Epicatechin-hexoside |
| **19** | 9.16 | 289 | 305 | 219(45),179(41),125(100) | Epigallocatechin |
| **20** | 9.86 | 288 | 289 | 245(100) | (-)-Epicatechin |
| **21** | 10.04 | 312 | 337 | 173(100),163(8) | Shikimic acid-*O*-hexoside |
| **22** | 10.63 | 281 | 865 | 451(44),425(59),407(97), 289(65) | Procyanidin trimer isomer I |
| **23** | 11.93 | 359 | 547 | 253(100) | Chrysin-6-*C*-pentoside-8-*C*-glucoside |
| **24** | 12.37 | 326 | 437 | 341(100),179(31) | Methyl-sulfate caffeic acid hexoside |
| **25** | 12.54 | 286 | 1153 | 865(22), 713(4), 577(33), 575(16), 561(20), 289(100) | Procyanidin tetramer isomer II |
| **26** | 13.85 | 288 | 1441 | 1153(27),865(92),577(47),289(25),287(10) | Procyanidinn pentamer isomer I |
| **27** | 14.29 | 280 | 865 | 451(44),425(59),407(97), 289(65) | Procyanidin trimer isomer II |
| **28** | 14.87 | 281 | 865 | 451(44),425(59),407(97), 289(65) | Procyanidin trimer isomer III |
| **29** | 14.97 | 315 | 425 | 263(100),161(12),159(21) | 3-*O*-methyl gallic acid sulfate-hexoside |
| **30** | 15.21 | 281 | 1441 | 1153(27),865(92),577(47),289(25),287(10) | Procyanidinn pentamer isomer II |
| **31** | 15.45 | 330 | 595 | 287(100),269(31) | Eriodictyol-rutinoside |
| **32** | 15.56 | 282 | 1153 | 865(22), 713(4), 577(33), 575(16), 561(20), 289(100) | Procyanidin tetramer isomer III |
| **33** | 16.14 | 288 | 865 | 451(44),425(59),407(97), 289(65) | Procyanidin trimer isomer IV |
| **34** | 17.06 | 353 | 623 | 315(100) | Isorhamnetin-3-*O*-deoxyhexosyl-hexoside |
| **35** | 17.66 | 609 | 355 | 301(100) | Quercetin-3-*O-*(6-*O*-rhamnosyl)-galactoside |
| **36** | 17.91 | 609 | 354 | 301(100) | Quercetin-3-*O*-rutinoside |
| **37** | 19.16 | 609 | 352 | 301(100) | Quercetin-3-*O*-glucoside |
| **38** | 19.82 | 347 | 593 | 285(100) | Kaempferol-3-*O*-(6-*O-*rhamnosyl)-glucoside |
| **39** | 21.21 | 347 | 593 | 285(100) | Kaempferol-3-*O*-rutinoside |
| **40** | 22.15 | 353 | 623 | 315(100) | Isorhamnetin-3-*O-*rutinoside |
| **41** | 22.77 | 447 | 348 | 301(100) | Quercetin-3-*O*-rhamnoside |
| **42** | 28.95 | 320 | 565 | 521(100),381(31),311(15),193(38) | Acetogenin isomer I |
| **43** | 29.48 | 320 | 565 | 521(100) | Acetogenin isomer II |
| **44** | 30.12 | 320 | 565 | 521(100) | Acetogenin isomer III |

**Table SM2** **⏐** Phenolic compounds tentatively identified in the leaves, stem bark, and seeds of *A. squamosa* hydroethanolic extracts, decoction and infusion preparations. The retention time (Rt), wavelength of maximum absorption (λmax) in the UV-Vis region, and mass spectral data are presented.

| **Peaks** | **Rt (min)** | **λmax (nm)** | **[M-H]^-^ *m/z*** | | **MS^2^ (*m/z*)** | **Tentative Identification** |
| --- | --- | --- | --- | --- | --- | --- |
| **1** | 4.31 | 324 | 209 | 191(100) | | Glucaric acid |
| **2** | 4.52 | 276 | 341 | 179(100) | | Caffeic acid-hexoside |
| **3** | 4.69 | 321 | 391 | 217(100),179(55),135(8) | | Dihydrocaffeic acid-glucoronide |
| **4** | 5.13 | 312 | 315 | 163(100) | | Galloyl-*p*-coumaric acid |
| **5** | 5.43 | 313 | 341 | 305(100),169(23) | | Dihydroxygallocatechin |
| **6** | 5.77 | 281 | 1153 | 865(22), 713(4), 577(33), 575(16), 561(20), 289(100) | | Procyanidin tetramer isomer I |
| **7** | 6.05 | 313 | 355 | 191(100) | | Ferulic acid-*O*-hexoside |
| **8** | 6.13 | 305 | 191 | 173(100),127(12) | | Quinic acid |
| **9** | 6.41 | 281 | 577 | 451(38),425(67),407(100),289(76) | | Procyanidin dimer isomer II |
| **10** | 6.73 | 322 | 353 | 191(32),179(52)173(100) | | 4-*O*-caffeoylquinic acid |
| **11** | 7.24 | 288 | 289 | 245(100) | | (+)-Catechin |
| **12** | 7.28 | 312 | 355 | 191(100) | | Ferulic acid-6-*C*-hexoside |
| **13** | 7.34 | 308 | 565 | 271(100) | | Naringenin-hexoside-pentoside |
| **14** | 8.02 | 288 | 577 | 451(38),425(67),407(100),289(76) | | Procyanidin dimer isomer IV |
| **15** | 8.72 | 295 | 431 | 289(100) | | (-)-Epicatechin-hexoside |
| **16** | 9.16 | 289 | 305 | 219(45),179(41),125(100) | | Epigallocatechin |
| **17** | 9.86 | 288 | 289 | 245(100) | | (-)-Epicatechin |
| **18** | 10.04 | 312 | 337 | 173(100),163(8) | | Shikimic acid-*O*-hexoside |
| **19** | 10.63 | 281 | 865 | 451(44),425(59),407(97), 289(65) | | Procyanidin trimer isomer I |
| **20** | 11.93 | 359 | 547 | 253(100) | | Chrysin-6-*C*-pentoside-8-*C*-glucoside |
| **21** | 12.37 | 326 | 437 | 341(100),179(31) | | Methyl-sulfate caffeic acid hexoside |
| **22** | 13.85 | 288 | 1441 | 1153(27),865(92),577(47),289(25),287(10) | | Procyanidinn pentamer isomer I |
| **23** | 14.97 | 315 | 425 | 263(100),161(12),159(21) | | 3-*O*-methyl gallic acid sulfate-hexoside |
| **24** | 15.45 | 330 | 595 | 287(100),269(31) | | Eriodictyol-rutinoside |
| **25** | 15.56 | 282 | 1153 | 865(22), 713(4), 577(33), 575(16), 561(20), 289(100) | | Procyanidin tetramer isomer III |
| **26** | 17.06 | 353 | 623 | 315(100) | | Isorhamnetin-3-*O*-deoxyhexosyl-hexoside |
| **27** | 17.66 | 609 | 355 | 301(100) | | Quercetin-3-*O*-(6-*O*-rhamnosyl)-galactoside |
| **28** | 17.91 | 609 | 354 | 301(100) | | Quercetin-3-*O*-rutinoside |
| **29** | 19.16 | 463 | 352 | 301(100) | | Quercetin-3-*O*-glucoside |
| **30** | 19.82 | 347 | 593 | 285(100) | | Kaempferol-3-*O*-(6-*O-*rhamnosyl)-glucoside |
| **31** | 21.21 | 347 | 593 | 285(100) | | Kaempferol-3-*O-*rutinoside |
| **32** | 22.15 | 353 | 623 | 315(100) | | Isorhamnetin-3-*O*-rutinoside |
| **33** | 22.77 | 447 | 348 | 301(100) | | Quercetin-3-*O*-rhamnoside |
| **34** | 25.67 | 223,276 | 773 | 729(100),685(25),369(11) | | Acetogenin isomer I |
| **35** | 26.61 | 223,279 | 847 | 803(100),739(65),555(20),255(8) | | Acetogenin isomer II |
| **36** | 26.91 | 223,279 | 847 | 803(100),739(65),555(20),255(8) | | Acetogenin isomer III |
| **37** | 31.61 | 223,277 | 835 | 809(100),565(49),355(21) | | Acetogenin isomer IV |
| **38** | 33.64 | 223,279 | 831 | 787(100),621(51),465(12),397(11),315(6) | | Acetogenin isomer V |
| **39** | 34.49 | 223,278 | 999 | 969(100),869(25),763(39),383(8) | | Acetogenin isomer VI |

**Table SM3 ⏐** Phenolic compounds tentatively identified in the leaves, stem bark, and seeds of *A. senegalensis* hydroethanolic extracts, decoction and infusion preparations. The retention time (Rt), wavelength of maximum absorption (λmax) in the UV-Vis region, and mass spectral data are presented.

| **Peaks** | **Rt (min)** | **λmax (nm)** | **[M-H]^-^ *m/z*** | **MS^2^ (*m/z*)** | **Tentative Identification** |
| --- | --- | --- | --- | --- | --- |
| **1** | 4.31 | 324 | 209 | 191(100) | Glucaric acid |
| **2** | 4.69 | 321 | 391 | 217(100),179(55),135(8) | Dihydrocaffeic acid-glucoronide |
| **3** | 5.13 | 312 | 315 | 163(100) | Galloyl-*p*-coumaric acid |
| **4** | 5.43 | 313 | 341 | 305(100),169(23) | Dihydroxygallocatechin |
| **5** | 5.48 | 280 | 577 | 451(38),425(67),407(100),289(76) | Procyanidin dimer isomer I |
| **6** | 5.69 | 311 | 355 | 193(100) | Ferulic acid-hexoside |
| **7** | 5.77 | 281 | 1153 | 865(22), 713(4), 577(33), 575(16), 561(20), 289(100) | Procyanidin tetramer isomer I |
| **8** | 6.13 | 305 | 191 | 173(100),127(12) | Quinic acid |
| **9** | 6.41 | 281 | 577 | 451(38),425(67),407(100),289(76) | Procyanidin dimer isomer II |
| **10** | 6.44 | 325 | 337 | 163(100),119(11) | *p*-coumaroylquinic acid |
| **11** | 6.73 | 322 | 353 | 191(32),179(52)173(100) | 4-*O*-caffeoylquinic acid |
| **12** | 7.24 | 288 | 289 | 245(100) | (+)-Catechin |
| **13** | 7.34 | 308 | 565 | 271(100) | Naringenin-hexoside-pentoside |
| **14** | 7.39 | 322 | 353 | 191(100),179(15),135(8) | 5-*O-*caffeoylquinic acid |
| **15** | 7.61 | 287 | 577 | 451(38),425(67),407(100),289(76) | Procyanidin dimer isomer III |
| **16** | 8.02 | 288 | 577 | 451(38),425(67),407(100),289(76) | Procyanidin dimer isomer IV |
| **17** | 9.16 | 289 | 305 | 219(45),179(41),125(100) | Epigallocatechin |
| **18** | 9.86 | 288 | 289 | 245(100) | (-)-Epicatechin |
| **19** | 10.63 | 281 | 865 | 451(44),425(59),407(97), 289(65) | Procyanidin trimer isomer I |
| **20** | 11.93 | 359 | 547 | 253(100) | Chrysin-6-*C*-pentoside-8-*C*-glucoside |
| **21** | 12.54 | 286 | 1153 | 865(22), 713(4), 577(33), 575(16), 561(20), 289(100) | Procyanidin tetramer isomer II |
| **22** | 13.85 | 288 | 1441 | 1153(27),865(92),577(47),289(25),287(10) | Procyanidinn pentamer isomer I |
| **23** | 14.29 | 280 | 865 | 451(44),425(59),407(97), 289(65) | Procyanidin trimer isomer II |
| **24** | 14.87 | 281 | 865 | 451(44),425(59),407(97), 289(65) | Procyanidin trimer isomer III |
| **25** | 15.21 | 281 | 1441 | 1153(27),865(92),577(47),289(25),287(10) | Procyanidinn pentamer isomer II |
| **26** | 15.45 | 330 | 595 | 287(100),269(31) | Eriodictyol-rutinoside |
| **27** | 15.56 | 282 | 1153 | 865(22), 713(4), 577(33), 575(16), 561(20), 289(100) | Procyanidin tetramer isomer III |
| **28** | 16.14 | 288 | 865 | 451(44),425(59),407(97), 289(65) | Procyanidin trimer isomer IV |
| **29** | 17.06 | 353 | 623 | 315(100) | Isorhamnetin-3-*O*-deoxyhexosyl-hexoside |
| **30** | 17.66 | 609 | 355 | 301(100) | Quercetin-3-*O*-(6-*O*-rhamnosyl)-galactoside |
| **31** | 17.91 | 609 | 354 | 301(100) | Quercetin-3-*O*-rutinoside |
| **32** | 19.16 | 463 | 352 | 301(100) | Quercetin-3-*O*-glucoside |
| **33** | 19.82 | 347 | 593 | 285(100) | Kaempferol-3-*O*-(6-*O*-rhamnosyl)-glucoside |
| **34** | 21.21 | 347 | 593 | 285(100) | Kaempferol-3-*O*-rutinoside |
| **35** | 21.59 | 433 | 348 | 301(100) | Quercetin-3-*O*-pentoside |
| **36** | 22.15 | 353 | 623 | 315(100) | Isorhamnetin-3-*O-*rutinoside |
| **37** | 22.77 | 447 | 348 | 301(100) | Quercetin-3-*O*-rhamnoside |


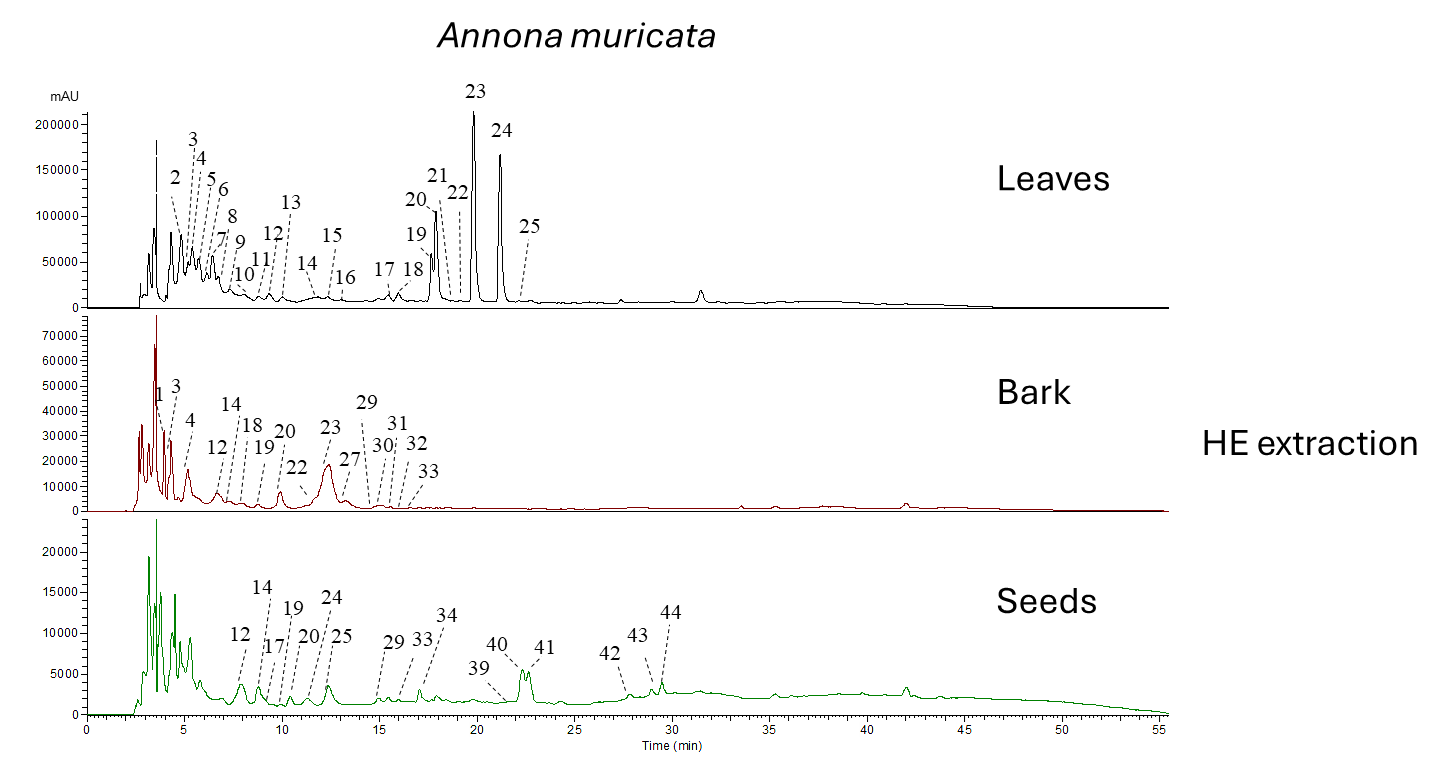


A

B

CC

**Figure S1** **⏐** Phenolic profile of leaves (A), bark (B), and seeds (C) from *A. muricata* hydroethanolic extracts. The peaks are identified in Table SM1.


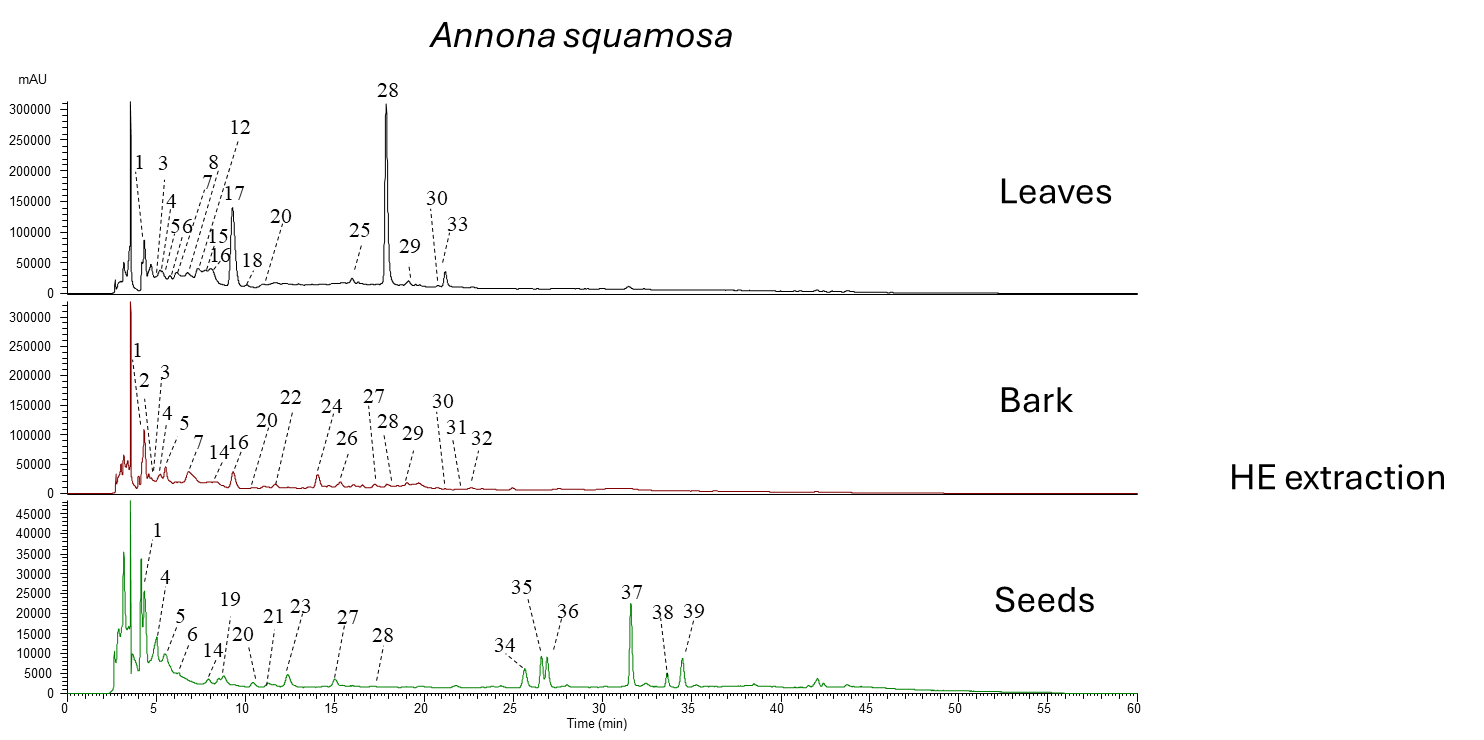


A

B

CC

**Figure S2 ⏐** Phenolic profile of leaves (A), bark (B), and seeds (C) from *A. squamosa* hydroethanolic extracts. The peaks are identified in Table SM2.


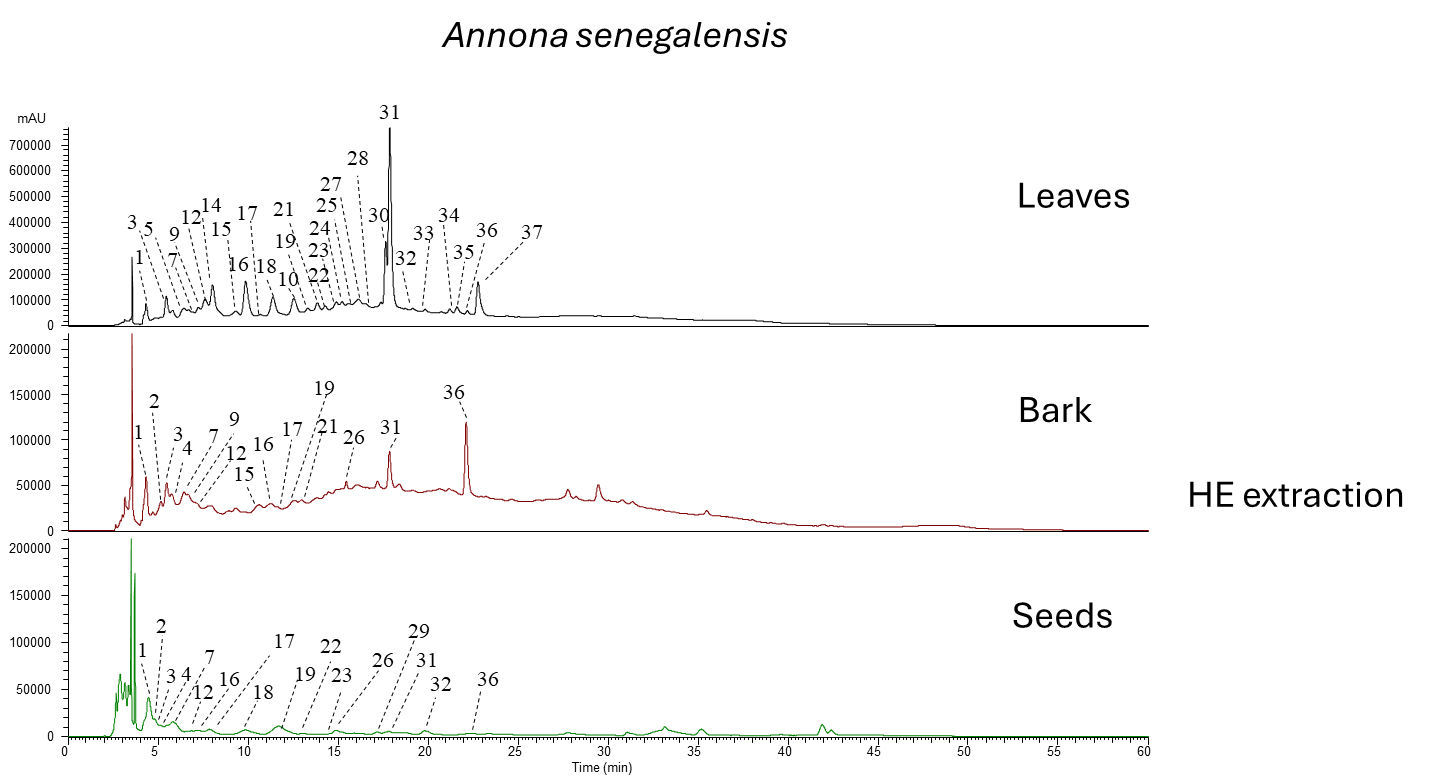


A

B

CC

**Figure S3 ⏐** Phenolic profile of leaves (A), bark (B), and seeds (C) from *A. senegalensis* hydroethanolic extracts. The peaks are identified in Table SM3.
